# ACSL4-mediated lipid rafts prevent membrane rupture and inhibit immunogenic cell death in melanoma

**DOI:** 10.1038/s41419-024-07098-3

**Published:** 2024-09-29

**Authors:** Xi Zhao, Zenglu Zhao, Bingru Li, Shuyu Huan, Zixi Li, Jianlan Xie, Guoquan Liu

**Affiliations:** 1 State Key Laboratory of Natural and Biomimetic Drugs, School of Pharmaceutical Sciences, Peking University, Beijing, China; 2 Department of Pharmaceutical Analysis, School of Pharmaceutical Sciences, Peking University, Beijing, China; 3 Department of Pathology, Beijing Friendship Hospital, Capital Medical University, Beijing, China; 4 Department of Biomedical Engineering, Institute of Advanced Clinical Medicine, Peking University, Beijing, 100191 China

**Keywords:** Cancer microenvironment, Cancer metabolism, Chemotherapy

## Abstract

Chemotherapy including platinum-based drugs are a possible strategy to enhance the immune response in advanced melanoma patients who are resistant to immune checkpoint blockade (ICB) therapy. However, the immune-boosting effects of these drugs are a subject of controversy, and their impact on the tumor microenvironment are poorly understood. In this study, we discovered that lipid peroxidation (LPO) promotes the formation of lipid rafts in the membrane, which mediated by Acyl-CoA Synthetase Long Chain Family Member 4 (ACSL4) impairs the sensitivity of melanoma cells to platinum-based drugs. This reduction primarily occurs through the inhibition of immunogenic ferroptosis and pyroptosis by reducing cell membrane pore formation. By disrupting ACSL4-mediaged lipid rafts via the removal of membrane cholesterol, we promoted immunogenic cell death, transformed the immunosuppressive environment, and improved the antitumor effectiveness of platinum-based drugs and immune response. This disruption also helped reverse the decrease in CD8^+^ T cells while maintaining their ability to secrete cytokines. Our results reveal that ACSL4-dependent LPO is a key regulator of lipid rafts formation and antitumor immunity, and that disrupting lipid rafts has the potential to enhance platinum-based drug-induced immunogenic ferroptosis and pyroptosis in melanoma. This novel strategy may augment the antitumor immunity of platinum-based therapy and further complement ICB therapy.

## Background

Immune checkpoint blockade (ICB) is a frequently used first-line treatment in patients with metastatic melanoma [1]. However, almost 25% of responding patients acquire resistance to PD-1 inhibitors within 2 years of treatment [2]. Objective responses and durable benefits from anti–cytotoxic T-lymphocyte antigen 4 antibodies occur in only 15–30% of patients [3, 4]. Overcoming resistance to anti-ICB therapy is currently one of the major challenges in cancer immunotherapy and oncology [5].

Recently, enhancing the therapeutically responsive ratio of ICB combination treatment has attracted hot interest in cancer immunotherapy [6–8]. Cytotoxic drugs, including platinum-based drugs, have been widely explored preclinically and clinically to boost the effect of ICB [9, 10]. Although quite a few therapies have been approved by the FDA [11–13], the rationale for the combined use of these drugs in immunotherapy remains controversial [14]. Dual effects on the immune system have been reported due to their usually poor selectivity and adverse reactions. For instance, platinum-based drugs are reported to enhance tumor immunogenicity by increasing antigen presentation and T-cell infiltration, mainly through triggering immunogenic cell death (ICD) of tumor cells [15–17]. However, they may also negatively modulate the immune system through myelosuppression, a common side effect correlated with platinum-based drugs [18–20].

The ICD effects of cytotoxic drugs, characterized by the release of damage-associated molecular patterns (DAMPs) and tumor-specific antigens, rely largely on the pathways by which cell death is executed [21]. Although platinum-based drugs mainly induce apoptosis by interfering with the replication and transcription of DNA, they can trigger multiple other cell death pathways in a context-dependent manner [22]. Among these complicated cell death pathways, the immunogenicity of ferroptosis and pyroptosis has been well demonstrated and investigated in depth [23–26]. Platinum drugs can enhance lipid peroxidation (LPO) and lead to ferroptosis by defuncting the GSH-GPX4 system [27, 28]. They can also activate Gasdermin-E (GSDME) or GSDMD after cleavage of Caspase-3 and steer apoptotic cell death to pyroptotic cell death in some cancer cells [29–31]. Therefore, enhancing the ratio of ferroptosis and pyroptosis in cell death pathways provides a potential strategy to amplify the ICD effect of platinum-based therapy.

The tumor microenvironment (TME) is critical to the efficacy of many antitumour therapies through inducing resistance to small molecule drugs and anti-ICB antibodies [32]. In particular, we have shown that abnormal cholesterol metabolism in the TME protects cancer cells from ferroptosis [33]. The protective role of cholesterol was ascribed to the formation of lipid rafts, which are dynamic domains enriched in cholesterol and glycol-lipids. These lipid rafts are formed concomitantly under oxidative stress and abundant cholesterol and can enhance the resistance of cancer cells to elevated LPO. Ferroptosis is characterized by membrane disruption [34], which is also shared by pyroptosis, where pore-forming gasdermin causes dramatic membrane rupture [35]. Based on their similarities, we hypothesize that lipid rafts may also attenuate the progression of pyroptosis. Here, we examined our hypothesis in aggressive melanoma, where platinum-based drugs have been widely used in many countries [20, 36]. We found that LPO-promoted lipid rafts significantly reduced the efficacy of platinum-based drugs by inhibiting both ferroptotic and pyroptotic cell death. Disruption of lipid rafts sensitizes cells to these two cell death modalities and enhances the ICD effects of platinum-based drugs in vitro. Moreover, this strategy significantly increased the ratio of CD8^+^ T cells as well as their ability to secrete cytokines in vivo. Our studies demonstrate that lipid rafts are a potentially new target to improve the immunosuppressive microenvironment, which may help overcome the resistance to ICB therapy in melanoma.

## Results

### Lipid rafts correlate with survival in melanoma

**Fig. 1:**
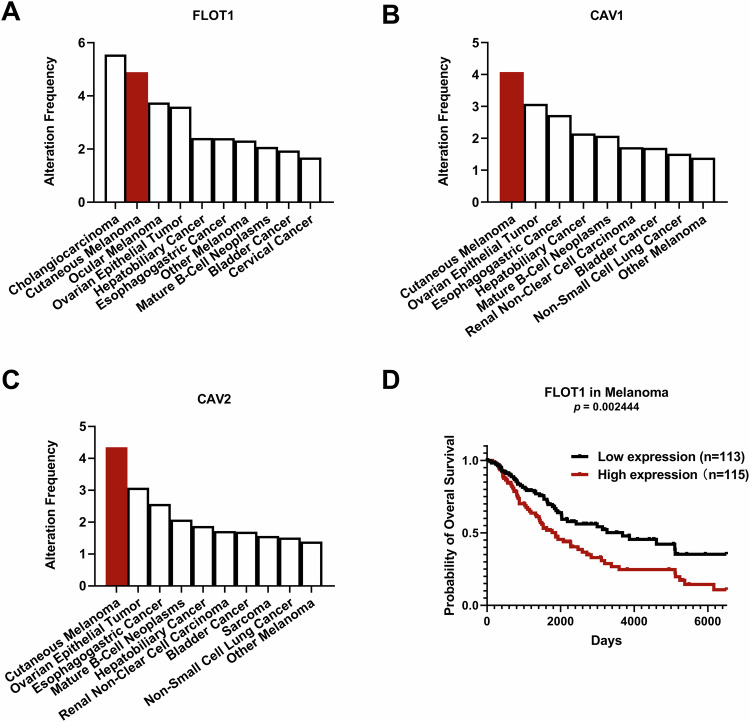
Higher levels of lipid rafts correlate with worse survival in melanoma. **A**–**C** Among the 32 types of cancer, the top 10 cancers with amplification alteration frequencies were FLOT1, CAV1, and CAV2. **D** Higher levels of FLOT1 (lipid raft) expression correlate with worse survival in melanoma cancer patients. Log-rank *P* value for Kaplan‒Meier plot showing results from analysis of correlation between protein expression level and patient survival. **A**–**D** Large-scale cancer genomics data sets were mined from TCGA and visualized by cBioPortal.

Caveolins (CAVs) and flotillins (FLOTs) are typical components of membrane lipid rafts and essential regulators of lipid raft-mediated cancer progression. It has been reported that the amplification mutation of these lipid raft-related proteins affects cancer cell invasion and modulates the tumor immune microenvironment and the response to immunotherapy [37, 38]. In particular, among the 32 types of cancer species from 50 studies involving 13679 people (http://cbioportal.org), we found that the amplification mutations of CAV1, CAV2, and FLOT1 were primarily singled out in cutaneous melanoma (Fig. 1A–C). Consistently, data mined from TCGA consistently show that a higher level of lipid raft (FLOT1) expression correlates with worse survival in melanoma (Fig. 1D). Therefore, we speculated that lipid rafts are a potential biomarker and therapeutic target for melanoma.

### ACSL4-dependent LPO drives the formation of lipid rafts

**Fig. 2:**
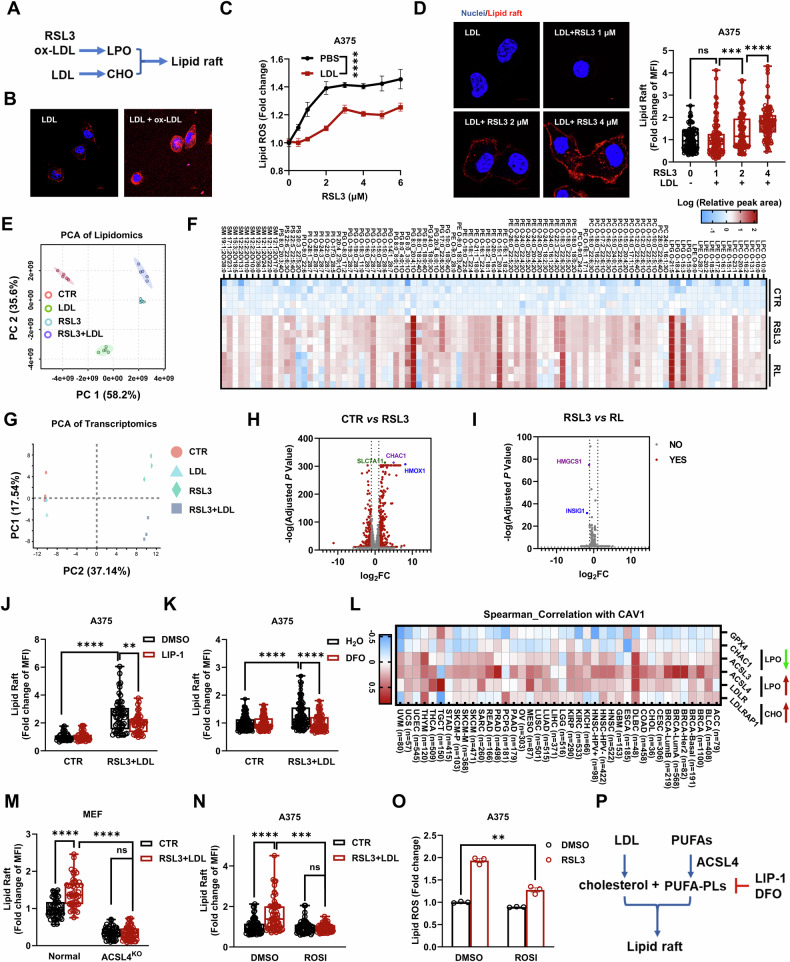
ACSL4 is required for LPO to induce lipid rafts formation. **A** A simple diagram illustrating the process by which cholesterol promotes the formation of lipid rafts in an oxidative environment. **B** Effect of ox-LDL (20 μg/mL) on lipid rafts in A375 cells. Scale bars = 10 μm. **C** Effect of LDL (40 μg/mL) on the LPO assayed by C11-BODIPY in RSL3-treated A375 cells. The results are derived from 3 independent replicates. **D** Dependence of lipid rafts on the RSL3 concentration in A375 cells. Scale bars = 10 μm. **E** Principal component analysis (PCA) of lipidomics shows slight separation of the RLS3 and RL groups. **F** Lipidomics analysis of A375 cells treated with RSL3 (2 μM) or RSL3 plus LDL (40 μg/mL) for 24 h. The heatmap shows that LDL does not decrease oxidized phospholipids and sphingomyelin. Measurements were performed with 5 replicates. **G**. PCA of transcriptomics. **H**, **I** Volcano plot showing the changes in gene expression in A375 cells treated with RSL3 (2 μM) or RSL3 plus LDL (40 μg/mL). *P*_adjusted_ < 0.05 and FC < 0.5 or >2 were considered significant changes (red dots). No significant differences were black. Statistical significance was assessed using DESeq2. *P*-adjusted < 0.05 was considered statistically significant (**H**, **I**). **J**, **K** Effect of LIP-1 (2 μM, **J**) and DFO (50 μM, **K**) on lipid rafts. **L** Correlation between CAV1 and LPO-related genes, including GPX4, CHAC1, ACSL3, ACSL4, LDLR and LDLRAP1, in 40 types of cancer from TIMER 2.0 [41]. **M** Effect of rosiglitazone (ROSI, 50 μM) on lipid rafts in A375 cells. **N** Lipid peroxidation in A375 cells treated with DMSO, RSL3, or ROSI (50 μM). **D**, **J**, **K**, **M**–**N** Each data point represents an individual cell, with ~50–100 cells randomly counted for statistical analysis. **O** Effect of ACSL4^KO^ on lipid rafts in MEFs. Each data point represents an independent repeat. **P** A schematic diagram illustrating that cholesterol and PUFAs jointly induce lipid rafts in an ACSL4-dependent manner. Statistical significance was assessed using two-way ANOVA (**C**) or an unpaired two-tailed t test (**D**, **J**, **K**, **M**–**O**).

The cell membrane is a mixture of liquid-ordered (Lo) domains, often referred to as lipid rafts, and liquid-disordered (Ld) domains. To increase the content of lipid rafts, we initially supplemented cells with low-density lipoprotein (LDL), which is the main carrier of exogeneous cholesterol. However, LDL alone did not promote the formation of lipid rafts (Fig. S1A). We previously reported that accumulated cholesterol in the TME protected cancer cells from LPO and ferroptosis both in vitro and in vivo [33]. Moreover, elevated LPO, triggered either by Oxidized LDL (ox-LDL) or inhibiting GPX4 by RSL3, could promote the formation of lipid rafts in the presence of LDL [33]. Therefore, we attempted to disrupt the balance between the Lo and Ld domains by adding ox-LDL as a source of LPO, which is usually enriched in the TME. Experimental data demonstrated that the concomitant presence of ox-LDL and LDL significantly promoted lipid raft formation (Fig. 2A, B). Besides, we studied the effects of LDL, ox-LDL, or RLS3 on cellular cholesterol levels after a recovery period of 24 h. While treatment with LDL alone did not significantly increase the cellular cholesterol, treatment with RSL3 or ox-LDL maintained the higher cholesterol levels in combination with LDL (Fig. S1B, C). These findings suggest that LPO contributes to maintain elevated levels of cholesterol, a main component of lipid rafts.

To further clarify their correlation, the LPO triggered by RSL3 was assayed through C11-Bodipy fluorescence, while the lipid rafts were quantified by GM1 staining. In the regime of low-dose RSL3 challenge (e.g., 1 μM), LPO was nearly suppressed by LDL, and no elevated content of lipid rafts was observed. When the dose of RSL3 increased, the LPO level was elevated despite an obvious inhibitory effect exerted by LDL. Correspondingly, a clear dose-dependent effect of lipid rafts on RSL3 was also observed (Fig. 2C, D). We then focused on the medium regime where significant lipid rafts were formed (RSL3 = 2 μM) and performed lipidomic and transcriptomic analysis. In the lipidomic analysis, oxidized phospholipids featuring LPO were widely identified in RL-treated cells (both RSL3 and LDL were added), which was comparable to RSL3-treated cells (Fig. 2E, F). Transcriptomics also showed that RL treatment resulted in higher RNA levels of LPO-related genes, including HMOX-1 [39] and CHAC1 [40] (Fig. 2G, H), in a similar fashion to RSL3 treatment (Fig. 2I).

Furthermore, we modulated the LPO level using two ferroptosis inhibitors, Liproxstatin-1 (LIP-1, reducing lipid peroxides) and Deferoxamine (DFO, chelating irons). Both reduced the formation of lipid rafts, as expected (Figs. 2J, K, S1D, E). In data mined from TIMER 2.0 analysis [41], the expression of lipid rafts (CAV-1) correlated positively with con-LPO genes and cholesterol uptake genes but negatively with the pro-LPO gene GPX4 (Fig. 2L). In particular, the correlation of Acyl-CoA Synthetase Long Chain Family Member 4 (ACSL4), a key gene dictating cell sensitivity to ferroptosis, with lipid rafts was examined in MEFs. The increase in lipid rafts due to RL treatment was abrogated by ACSL4-knockout or inhibition using the small molecule rosiglitazone [42] (Figs. 2M, N, S1F–H). It has been reported that knockout of ACSL4 mainly reduced the peroxidation of PL-PUFAs, while the total LPO levels were not mitigated [43, 44]. Consistently, we found that no significant lipid rafts were formed in the ACSL4-knockout (or inhibited) cells, while the LPO assayed by C11-Bodipy fluorescence was elevated (Figs. 2O, S1I). We previously also observed that cytoplasmic ROS (e.g., triggered by chemo-drugs including cisplatin) could not promote the formation of lipid rafts [33]. These results together demonstrate that LPO has a specific and integral role in lipid raft formation, which is under the control of ACSL4 (Fig. 2P).

### ACSL4-mediated lipid rafts reduce the efficacy of platinum-based drugs

**Fig. 3:**
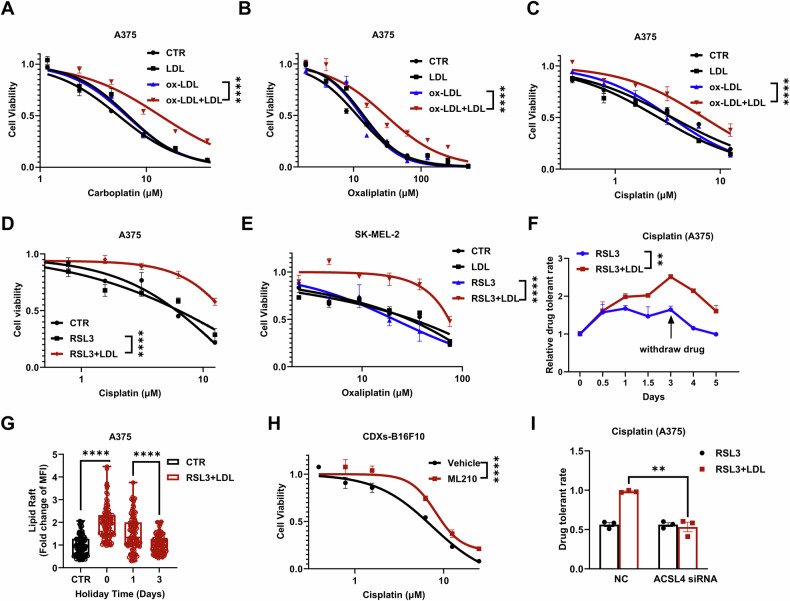
ACSL4-dependent LPO confers resistance to platinum-based drugs in melanoma cells. **A**–**E** Changes in A375 or SK-MEL-2 cell sensitivity to platinum-based drugs after RSL3 or RSL3 plus LDL (40 μg/mL) or ox-LDL (20 μg/mL) plus LDL treatment for 24 h. **F** Evaluation of the drug tolerance rate toward cisplatin in A375 cells treated with RSL3 (2 μM) or RSL3 plus LDL (40 μg/mL) for the indicated times. **A**–**F** The results are derived from 3 independent replicates. **G** Time evolution of lipid rafts in A375 cells after treatment with LDL and RSL3 for acquiring lipid rafts. Each data point represents an individual cell. Each data point represents an individual cell, with about 100 cells randomly counted for statistical analysis. **H** Role of ACSL4 siRNA in the drug resistance of A375 cells to cisplatin (25 μM) after 24 h of treatment with RSL3 (2 μM) or RSL3 plus LDL (40 μg/mL). The results are derived from 3 independent replicates. **I** The dose-dependent cytotoxicity of cisplatin on vehicle-treated xenograft-derived B16F10 cells or ML210-treated cells. Each data point represents an independent repeat. Statistical significance was assessed using two-way ANOVA (**A**–**E**, **I**) or an unpaired two-tailed t test (**G**, **H**).

Because of their clinical relevance in melanoma, we are interested in whether these lipid rafts affect the efficacy of platinum-based chemotherapy. A375 cells were first treated with ox-LDL and LDL (termed OL) for 24 h to stimulate the formation of lipid rafts (Fig. S2A). These cells were then exposed to platinum compounds (cisplatin, oxaliplatin or carboplatin), and significantly decreased sensitivity to these drugs was observed (Fig. 3A–C). Similar drug resistance was also observed in three other melanoma cell lines after acquiring lipid rafts, SK-MEL-2, SK-MEL-28, and B16F10 (Fig. S2B–E).

Consistently, when RSL3 was used to trigger LPO, only the combination of RSL3 and LDL treatment (termed RL) decreased the cellular sensitivity to platinum-based chemotherapy (Figs. 3D, E, S2F–L). This trend was similar to the formation of lipid rafts. To further demonstrate the correlation between lipid rafts and drug sensitivity, we monitored the time evolution of this drug resistance. The resistance to cisplatin increased over the incubation period with RL. When RL incubation was withdrawn after a 3-day treatment, the drug resistance started to diminish with recovery time (Fig. 3F). Simultaneously, the content of lipid rafts decreased following a similar trend (Figs. 3G, S3A). To demonstrate the relevance of lipid rafts in vivo, ML210 was applied to trigger LPO and the formation of lipid rafts in mouse xenografts. We then examined the sensitivity of those tumor cells detached from the xenografts. As expected, the ML210-treated xenograft-derived B16F10 cells exhibited lower sensitivity to platinum drugs than the vehicle-treated cells (Figs. 3H, S3B).

Moreover, knockdown of ACSL4, which prevents the formation of lipid rafts under RL treatment (Fig. 2M), abrogated the acquired resistance of melanoma cells to cisplatin (Fig. 3I). In summary, these results demonstrate the strong correlation between ACSL4-mediated lipid raft formation and platinum drug resistance.

### Lipid rafts inhibit ferroptosis and pyroptosis

**Fig. 4:**
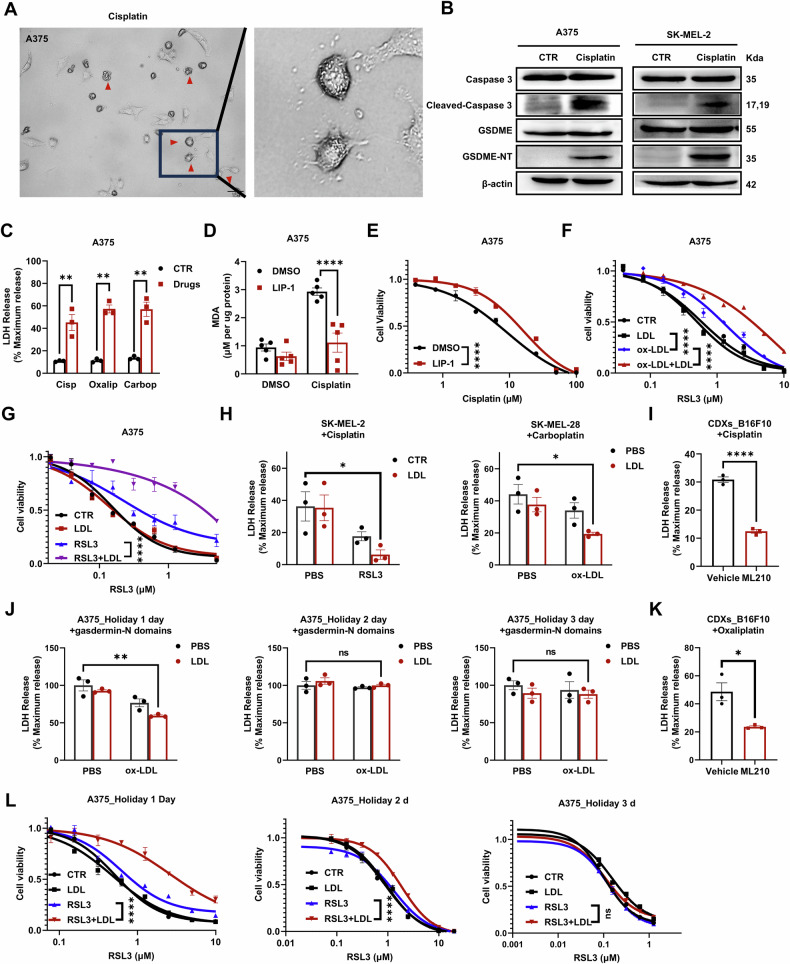
Lipid rafts reduce the efficacy of platinum-based drugs by inhibiting ferroptosis and pyroptosis in melanoma cells. **A** Representative images of inverted phase-contrast microscopy of pyrolytic A375 cells. The red triangle indicates that the cell is undergoing pyroptosis. Scale bars = 50 μm. **B** Cisplatin induces the activation of GSDME and caspase 3 in A375 and SK-MEL-2 cells, as measured by Western blotting. **C** Cisplatin, oxaliplatin, and carboplatin induced LDH release in A375 cells. **D** LIP-1 (2 μM) suppresses MDA content (measured by TBARS assay) triggered by cisplatin in A375 cells. **E** LIP-1 (2 μM) desensitizes A375 cells to cisplatin. **F**, **G** Changes in A375 cell sensitivity to RSL3 after the indicated pretreatment (LDL 40 μg/mL, ox-LDL 20 μg/mL, RLS3 2 μM, treated for 24 h). **H** Changes in LDH release induced by cisplatin or carboplatin after the indicated pretreatment in melanoma cell lines. **I**, **K** Decreased LDH release in A375 cells derived from ML210-treated xenografts. Time evolution of A375 cell sensitivity to gasdermin-N domain overexpression-induced pyroptosis (**J**) or RSL3-induced ferroptosis (**L**) after the indicated pretreatment. **C**, **D**, **H**–**K** Each data point represents an independent repeat. **E**–**G**, **L** The results are derived from 3 independent replicates. Statistical significance was assessed using two-way ANOVA (**E**–**G**, **L**) or an unpaired two-tailed t test (**C**, **D**, **H**–**K**).

Platinum-based anticancer drugs were reported to induce multiple cell death pathways, including pyroptosis, ferroptosis, and apoptosis, which were also confirmed in the three melanoma cell lines we investigated. First, platinum induced pyrolytic cellular swelling and activated the GSDME N-domain (Figs. 4A, B, S4, S5A), an executing protein of pyroptosis. Consistent with previous reports, the activation of GSDME N-domain was accompanied by the activation of Caspase 3 (Figs. 4B, S5A). The release of LDH resulting from membrane rupture in pyroptosis was also confirmed (Fig. 4C). Second, ferroptotic cell death was identified. MDA and 4-HNE, the decomposed derivatives of LPO, were significantly elevated after platinum treatment and were mitigated by the classic ferroptosis inhibitor (Figs. 4D, S5B). Cell viability was also partially rescued by LIP-1 (Figs. 4E, S5C). Third, the presence of apoptotic cell death was also demonstrated by the characteristic shrinkage of cellular nuclei (Fig. S5D).

We further examined whether ferroptotic and pyroptotic cell death could be blocked by lipid rafts. First, the OL-treated cells exhibited significant resistance to RSL3. The ox-LDL treatment imposed a minimal effect, while the effect of LDL was negligible (Fig. 4F). A similar resistance was also observed when RSL3 was used to trigger LPO (Figs. 4G, S6A). Second, LDH release was significantly reduced after OL or RL treatment (Figs. 4H, S6B). Again, individual treatment with ox-LDL or RSL3 had a much weaker effect. In the recovery experiments, the resistance to pyroptosis and ferroptosis also diminished with decreasing lipid rafts (Figs. 4J, L, S6C, D).

Moreover, in xenograft-derived B16F10 cells, ML210 treatment also lowered the release of LDH triggered by platinum drugs compared to vehicle treatment (Fig. 4I, K). In summary, these results demonstrate that lipid rafts reduce the sensitivity of melanoma cells to ferroptosis or pyroptosis triggered by platinum-based drugs.

### Lipid rafts protect membranes from pore formation

**Fig. 5:**
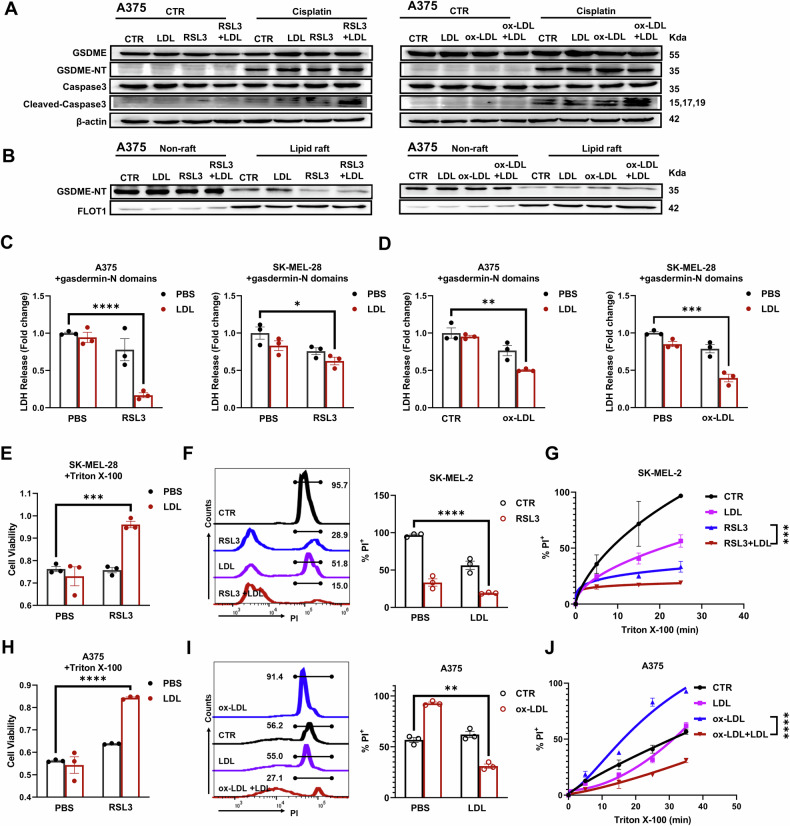
Lipid rafts suppress pyroptosis by reducing membrane pore formation. **A** RSL3 and LDL have no effect on cisplatin-induced activation of GSDME and caspase 3 in A375 cells, as measured by Western blotting. **B** The GSDME-N domain (membrane pore-forming protein) was enriched more in the nonraft domains than in the raft domains. **C**, **D** Decreased LDH release induced by gasdermin-N domain overexpression in cells pretreated with RSL3 plus LDL or ox-LDL plus LDL. Cell viability of SK-MEL-28 cells (**E**) or A375 cells (**H**) with the indicated pretreatment following Triton X-100 treatment. PI intensity and positive rate of SK-MEL-2 cells (**F**) or A375 cells (**I**) with the indicated pretreatment following Triton X-100 treatment examined by flow cytometry. Time-dependent membrane damage of Triton X-100 in SK-MEL-2 cells (**G**) or A375 cells (**J**) with the indicated pretreatment. **C**–**F**, **H**, **I** Each data point represents an independent repeat. **G**, **J** The results are derived from 3 independent replicates. Statistical significance was assessed using two-way ANOVA (**G**, **J**) or an unpaired two-tailed t test (**C**–**F**, **H**, **I**).

Cellular resistance to ferroptosis has been ascribed to decreasing membrane fluidity when lipid rafts are formed [33]. We were then dedicated to investigate how lipid rafts resist pyroptosis. Pyroptosis in melanoma cells was executed by cleavage of GSDME after activation of Caspase3 after treatment with platinum-based drugs. Interestingly, lipid rafts formed by either RL or OL had no effects on the expression of GSDME and did not prevent the cisplatin-induced cleavage of the GSDME-N domain and Caspase 3 (Fig. 5A). Cholesterol was reported to impair the pore formation function of GSDMD by modulating the membrane insertion and oligomerization of the GSDMD-N domain [45]. We, therefore, hypothesized that lipid rafts rich in cholesterol could resist pyroptosis by preventing the insertion of the GSDME-N domain in the plasma membrane. To prove our hypothesis, the lipid raft portion of the membranes was separated based on its resistance to non-ionic detergents. While lipid raft domains were confirmed by FLOT1, the GSDME-N domain was significantly enriched more in the non-raft domains than in the raft domains (Fig. 5B). This difference between the raft and non-raft domains was observed in all the treatments with either LDL, RSL3, Ox-LDL or their combination.

In addition to the GSDME-N domain triggered by cisplatin, we also overexpressed the GSDMD-N domain in A375 cells to examine the membrane protective effect of lipid rafts. The highly conserved N-domain of Gasdermin families, instead of the full-length proteins, induces extensive pyroptosis [35, 46]. LDH release experiments showed that both RL and OL treatments significantly lowered the membrane damage resulting from the pore formation of gasdermin-N domains. The effects of RSL3, ox-LDL, or LDL treatment alone were negligible (Fig. 5C, D). Moreover, the ionic detergent Triton X-100 was also used to directly lyse the cellular membrane, which was measured by cell viability and PI-positive cells. More cells survived the lysis of Triton X-100 after they acquired lipid rafts from either RL or OL treatment (Figs. 5E, H, S7A). The PI uptake measurements demonstrated the same trend that the RL and OL treatments outperformed the others, although the RSL3 and ox-LDL treatments differed from each other (Figs. 5F, G, I, J, S7B–E). Together, these results suggest that lipid rafts reduced membrane pore-formation and maintained structural integrity to resist pyroptosis.

### Disruption of lipid rafts promotes ferroptosis and pyroptosis in vitro

**Fig. 6:**
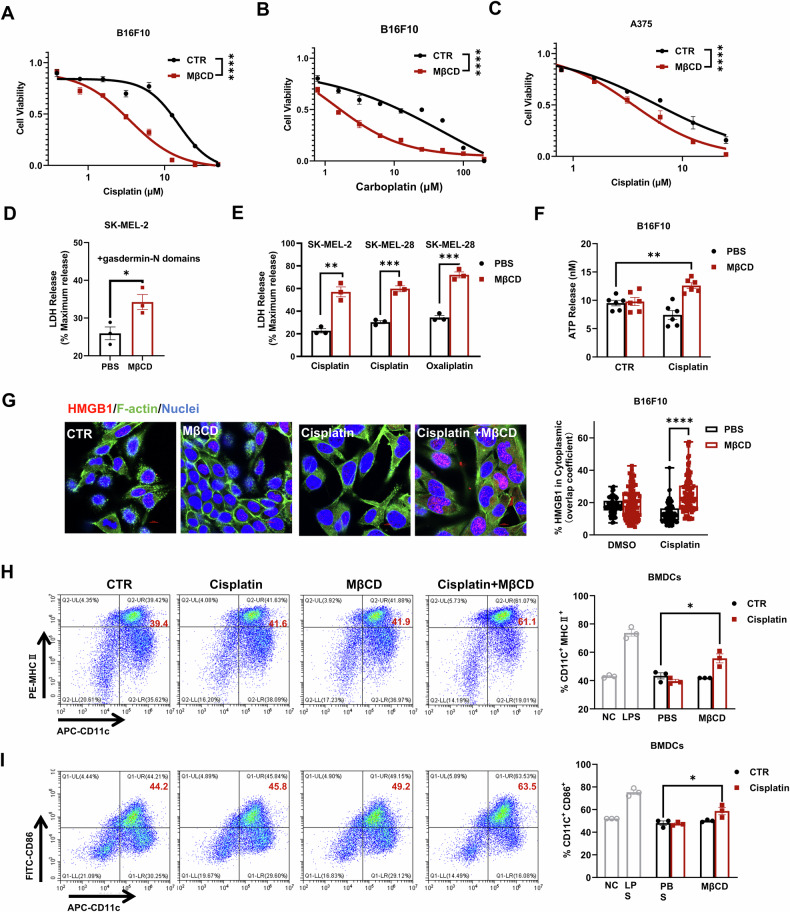
Lipid rafts are a promising target to enhance the immunogenicity of platinum-based drugs in vitro. Effect of MβCD on B16F10 (**A**, **B**) or A375 (**C**) cell sensitivity to cisplatin (**A**, **C**) or carboplatin (**B**). The results are derived from 3 independent replicates. Effect of MβCD on GSDME-NT overexpression-induced LDH release (**D**) or platinum-based drug-induced LDH release (**E**) in melanoma cells. **F** Effect of MβCD on cisplatin-induced LDH release in SK-MEL-2 cells. **E** Effect of MβCD on A375 cell sensitivity to cisplatin. **F** ATP release from B16F10 cells treated as indicated. **G** HMGB1 (red) translation from the nucleus to the cytoplasm was examined by immunofluorescence. Blue indicates DAPI-stained nuclei, green indicates phalloidin-stained F-actin. Scale bars = 10 μm. **H**, **I** B16F10 cells treated with the indicated treatments induce the maturation of BMDCs. LPS (100 ng/mL) was used as a positive control. **D**–**F**, **H**, **I** Each data point represents an independent repeat. **G** Each data point represents an individual cell. with ~100 cells randomly counted for statistical analysis. Statistical significance was assessed using two-way ANOVA (**A**–**C**) or an unpaired two-tailed t test (**D**–**I**).

Lipid rafts could be targeted by modulating cholesterol levels. MβCD, a cholesterol chelator in vitro and in vivo, can efficiently disrupt lipid rafts. It significantly increased the sensitivity of melanoma cells to cisplatin (Figs. 6A–C, S8A). Specifically, pyroptosis was increased based on elevated LDH release levels (Figs. 6D, E, S8B). Ferroptosis could also be promoted by MβCD in vitro and in a mouse xenograft model [33]. Both ferroptosis and pyroptosis have been found to be immunogenic due to the release of DAMPs [23–26]. Dying cancer cells from the combination treatment of MβCD and cisplatin released the highest amount of ATP (Fig. 6F). Furthermore, while MβCD alone could increase the expression of the high mobility group protein B1 (HMGB1), its combination with cisplatin afforded the most elevated level (Fig. 6G). In contrast, cisplatin itself slightly decreased the levels of released ATP and HMGB1 expression.

In vitro stimulation of murine bone marrow-derived dendritic cells (BMDCs) further demonstrated the boosting effects of MβCD in triggering ICD. Neither cisplatin- nor MβCD-treated cells could promote the maturation of BMDCs. Interestingly, those cells with the combination treatment resulted in significant maturation of more BMDCs, which was evidenced by the elevated expression levels of MHC II and CD86 in CD11c^+^ BMDCs (Fig. 6H, I). These in vitro results demonstrate that lipid rafts are a promising target to enhance the immunogenicity of platinum-based therapy.

### Disrupting lipid rafts amplifies the antitumour immunity of cisplatin in vivo

**Fig. 7:**
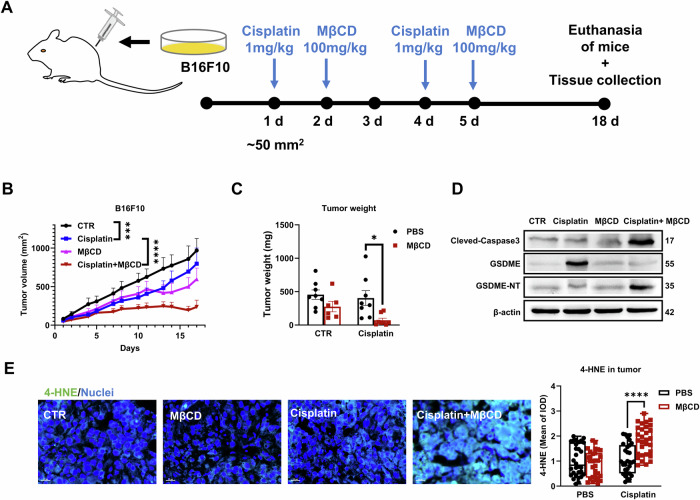
MβCD disrupting lipid rafts enhances ferroptosis and pyroptosis induced by cisplatin in vivo. **A** Schematic of tumor model establishment and subsequent treatment. **B**, **C** Growth curve and tumor weight of B16F10 xenografts with the indicated treatments. CTR = 8, MβCD = 6, Cisplatin = 8, MβCD + Cisplatin = 8. The weight of xenografts was obtained at the end of the indicated treatments. Each data point represents an individual xenograft tumor. **D** GSDME, GSDME-NT, and cleaved-caspase 3 protein analyzed by western blotting in B16F10 xenografts treated as indicated. **E** Immunofluorescence staining and quantification of 4-HNE-modified proteins (green) in paraffin-embedded xenografts at the end of the indicated treatment. Each data point represents a random field of view, with 5 tumor samples randomly selected per group. 3 slices was prepared from each tumor, spaced 50 μm apart, with a thickness of 5 μm. Two random fields of view were counted per slice. Blue indicates DAPI-stained nuclei. Scale bars = 20 μm. Statistical significance was assessed using two-way ANOVA (**B**) or an unpaired two-tailed *t* test (**C**, **E**).

To evaluate the in vivo effect of lipid raft disruption, we established a subcutaneous model with B16F10 cells (Fig. 7A). A low dose of cisplatin (1 mg/kg, usually ≥3 mg/kg for antitumour treatment [17, 29, 47, 48]) injected intraperitoneally exerted only a mild inhibitory effect on the growth of xenografts at the early stage, while its effect diminished at the advanced stage (Fig. 7B). The synergistic effect of MβCD with cisplatin to inhibit tumor growth became apparent 7 days after the start of medication (Fig. 7B).

Consistently, the tumor weight analysis at the end point showed that MβCD further enhanced the inhibitory effect of cisplatin (Figs. 7C, S9A). Fewer nuclei and more vacuoles were also identified in the haematoxylin-eosin (H&E) staining of tumor slices from the mice treated with cisplatin plus MβCD (Fig. S9B). TUNEL staining further highlighted that MβCD markedly promoted tumor cell death (Fig. S9C). Additionally, examination of LPO derivatives (4-HNE) showed that MβCD significantly enhanced the severity of LPO, indicating that more cancer cells underwent ferroptotic cell death (Fig. 7E).

The expression level of pyrolytic GSDME and its activator Cleaved-Caspase 3 was also increased in the combined treatment group (Fig. 7D). In contrast, cisplatin treatment did not show signs of elevated LPO or increased levels of activated caspase 3 and GSDME. These results confirmed the critical effect of MβCD in increasing ferroptosis and pyroptosis in melanoma cells in vivo after cisplatin treatment.

**Fig. 8:**
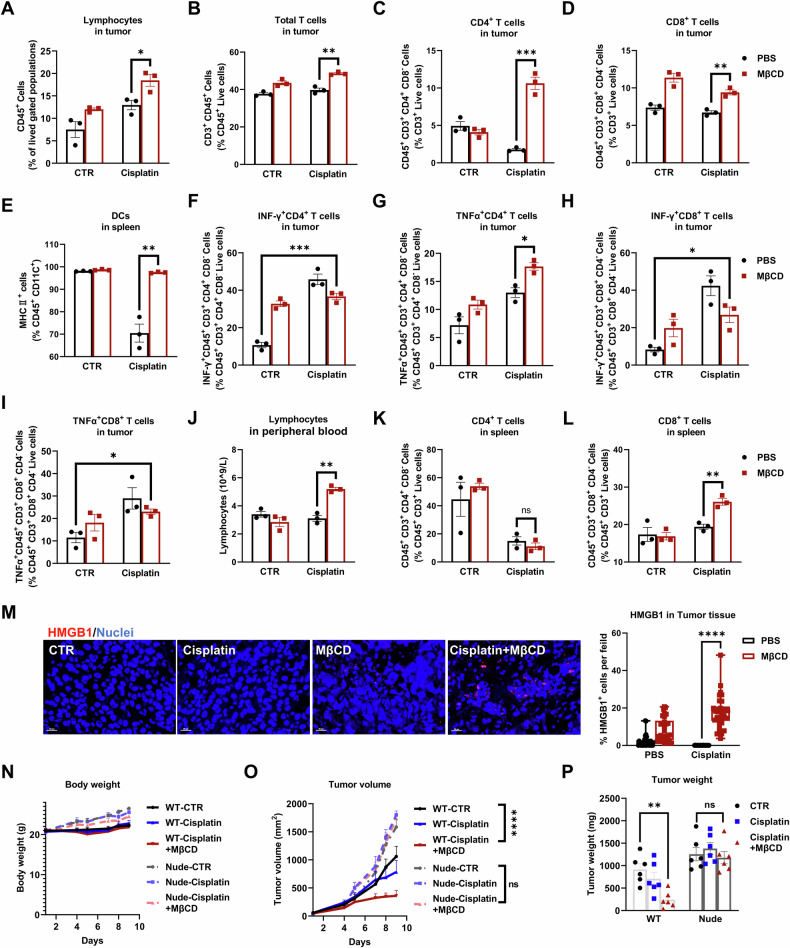
MβCD disrupting lipid rafts mediate T-cell activation against cisplatin-treated melanoma. **A**–**D** Infiltrating lymphocytes in xenografts at the end of the indicated treatment. **E** Percentages of MHCII^+^ DCs in the spleens of B16F10 xenografted mice with the indicated treatments. **F**–**I** Percentages of IFNγ^+^CD4^+^ (**F**), TNFα^+^CD4^+^ (**G**), IFNγ^+^CD8^+^ (**H**) or TNFα^+^CD8^+^ (**I**) T cells in B16F10 xenografts. **J** Lymphocyte counts in peripheral blood in B16F10 xenografted mice at the end of the indicated treatment. **K**, **L** Percentages of CD4^+^ or CD8^+^ T cells in the spleens of B16F10 xenografted mice with the indicated treatments. **M** Immunofluorescence staining and quantification of HMGB1 (red) in paraffin-embedded xenografts at the end of the indicated treatment. Blue indicates DAPI-stained nuclei. Scale bars = 20 μm. Each data point represents a random field of view, with 5 tumor samples randomly selected per group. 3 slices were prepared from each tumor, spaced 50 μm apart, with a thickness of 5 μm. Two random fields of view were counted per slice. **N** Weights of nude and wild-type mice with B16F10 xenografts with different treatments at different time points (days). **O**, **P** Growth curve and tumor weight of B16F10 xenografts in nude and wild-type mice with the indicated treatments. The weight of xenografts was obtained at the end of the indicated treatments. Each data point represents an individual xenograft tumor, each group consisted of 6 mice. **A**–**L** Each data point represents a tumor sample, 3 per group used for flow cytometric analysis. Statistical significance was assessed using two-way ANOVA (**O**) or an unpaired two-tailed t test (**A**–**M**, **P**).

According to previous reports, platinum-based drugs, including cisplatin, imposed contradictory effects on immune cells [47, 48], which was also observed in our studies. The ratio of tumor-infiltrating CD4^+^ and CD8^+^ T cells was decreased after treatment with a low dose of cisplatin (1 mg/kg), although the ratio of total lymphocytes and T cells was slightly increased (Fig. 8A–D). DCs in the spleen were also significantly decreased (Fig. 8E). In contrast, the activity of tumor-infiltrating CD4^+^ and CD8^+^ T cells was significantly enhanced by cisplatin, as evaluated by their ability to secrete cytokines, e.g., INF-γ and TNF-α (Fig. 8F–I). Interestingly, MβCD alone increased both the ratio and activity of tumor-infiltrating CD8^+^ T cells while increasing the activity of CD4^+^ T cells. These boosted adaptive immune activities may account for the tumor-inhibitory effects of MβCD.

We further studied the immunomodulatory effect of MβCD when combined with cisplatin. First, the number of lymphocytes and mature T cells in the TME, as well as the ratio of tumor-infiltrating CD8^+^ and CD4^+^ T cells, were all significantly increased compared with cisplatin treatment alone (Fig. 8A–D). Second, the activity of tumor-infiltrating CD4^+^ and CD8^+^ T cells was significantly enhanced by the combined treatment of MβCD and cisplatin, to a similar extent enhanced by cisplatin treatment alone (Fig. 8F–I). Third, lymphocytes in peripheral blood and CD8^+^ T cells in the spleen were also enhanced by the combined treatment of MβCD and cisplatin (Fig. 8J–L, Fig. S10B). Finally, MβCD recovered the cisplatin-induced DC decrease in mouse spleens (Fig. 8E), while MβCD alone had a negligible effect. Consistently, the highest amount of HMGB1 was observed in tumor slices treated with both cisplatin and MβCD (Fig. 8M). These results pointed to the immune-boosting effect of MβCD by enhancing the immunogenicity of cisplatin-induced cell death.

To further demonstrate the involvement of adaptive immunity in antitumour activity, immune-compromised nude mice were compared with normal mice. In these nude mice, cisplatin exhibited no effect on tumor growth, while a minimal inhibitory effect was observed in normal mice. Moreover, the significant inhibitory effect of MβCD and cisplatin was completely abrogated in nude mice (Fig. 8N–P). These results indicated that the synergetic effect of MβCD with cisplatin relied on the activity of T cells.

Anemia, nephrotoxicity, gastrointestinal injury and so on are common side effects in the clinical application of cisplatin [49]. Even under a low concentration of cisplatin (1 mg/kg), cisplatin still severely disrupted the crypts and villi of the small intestine (Fig. S11) and markedly induced intracellular vacuoles in the liver. Interestingly, these damages were attenuated by MβCD in the combined treatment mice (Fig. S11). The mouse spleens, lungs, kidneys and hearts all exhibited normal histological structures in the cisplatin combined with MβCD treatment group (Fig. S11). The weight of the spleens and the number of neutrophils did not differ among the groups (Fig. S12A, B). Blood biochemical analysis also suggested that the function of the heart, liver and kidney was normal for all mice (Fig. S12C–E). Moreover, cisplatin and MβCD reduced the weight of mice by less than 10% compared with that of control mice (Figs. 8N, S12F). Additionally, analysis of hemoglobin, glucose, and total cholesterol in peripheral blood confirmed that no mice were subject to malnutrition when treated with cisplatin and MβCD (Fig. S12G–I). Therefore, disruption of lipid rafts by MβCD enhanced the antitumour immune activity of cisplatin without increasing toxicity to normal tissues.

## Discussion

Despite their well-recognized antineoplastic effects, the immunological effects of cytotoxic agents, including platinum-based drugs, remain elusive. The occurrence of myelosuppression, a common side effect of chemotherapy [50], points to these drugs as an obvious detrimental factor to the immune system. Studies have shown that cisplatin can reduce the number of lymphocytes in the TME [47, 51]. On the other hand, especially as evidenced in vitro, the cytotoxicity of platinum-based drugs can trigger the release of DAMPs and tumor-specific antigens. These so-called ICDs may activate antigen-presenting DCs, which can further recruit and activate CD8^+^ T cells [21, 52]. Unsurprisingly, these contrary effects are intermingled in vivo to exert complex and context-dependent immunological phenotypes. In our melanoma xenograft mouse model, the decrease in TILs and spleen DCs agrees with the myelosuppression effect of cisplatin, even though the dose was kept minimal. However, a significant enhancement in the activity of TILs also indicates potential benefits of cisplatin treatment, either resulting from activation of antigen-presenting DCs or direct T-cell activation through stimulating chemokine secretion.

To enhance the efficacy of platinum-based drugs, the natural imperative is to maximize their immunological activation and mitigate damage to immune cells. Our study revealed that lipid rafts play a critical role in modulating the immunogenicity of platinum therapy. This role of lipid rafts is different from the previously known drug-resistant mechanisms in which downstream signaling pathways are modulated [53, 54]. We found that lipid rafts not only reduced the sensitivity of platinum-based drugs but also specifically protected melanoma cells from ferroptosis and pyroptosis, two cell pathways proven to be immunogenic [55–57]. The cell plasma membrane acquired resistance to pore-forming gasdermin proteins, as well as detergents, after the formation of lipid rafts [58]. Similar results have been previously reported in T cells, where increased membrane ordering (a feature shared by lipid rafts) helps resist the damage of pore-forming perforin [59]. Given the common presence of oxidative stress and cholesterol in the TME, lipid rafts are likely an unnoticed factor that also affects the immunological effect of platinum-based therapy. Therefore, targeting lipid rafts provides a novel strategy to amplify the immunogenicity of platinum-based therapy.

Depriving the cholesterol level from the membranes, e.g., by MβCD, is sufficient to enhance the immunogenicity of platinum-based therapy. It abrogated the acquired resistance of melanoma cells and enhanced the ratio of ferroptosis and pyroptosis. In a mouse model, we found that MβCD synergized with the antitumour effect of cisplatin. More importantly, it reversed the decreasing trend of TILs caused by cisplatin while retaining the high potential of TILs to secrete functional cytokines. Because of its biosafety as a pharmaceutic adjuvant, the combination of MβCD with platins exhibits good prospects in clinical translation. Interestingly, MβCD mitigated the obvious side effects of cisplatin on the intestine, which deserves further investigation. Moreover, MβCD could be readily modified with targeting groups to further improve its distribution in tumors.

We found that the formation of lipid rafts depends critically on ACSL4-dependent LPO, which revealed an interesting link between ferroptosis and pyroptosis. The oxidation of the PL-PUFAs, instead of the total PUFAs, promoted the formation of lipid rafts and subsequent drug resistance. This fact is consistent with current reports on the common critical role of the plasma membrane in the execution of ferroptosis and pyroptosis [60, 61]. Studies have shown that both ferroptosis and pyroptosis are harnessed by immune cells to confront malignant cells [57, 62]. Therefore, this ACSL4-dependent LPO, with its relevant pathways in ferroptosis, e.g., iron metabolism, provides a distinct perspective to understand the mechanism of immune evasion in cancers, as well as in viral infections.

## Materials and methods

### Chemicals

Cisplatin, Carboplatin, and Oxaliplatin were purchased from TargetMol. DFO were purchased from Sigma. RSL3, ML210, and liproxstatin-1 (LIP-1) were purchased from Selleck Chemicals, and MβCD and rosiglitazone (ROSI) were purchased from MedChemExpress. LDL and ox-LDL were obtained from Guangzhou Yiyuan Biological Technology Co., Ltd.

### Cell line culture

A375, B16F10, SK-MEL-2, SK-MEL-28, and MEF cells were purchased from the National Infrastructure of Cell Line Resource of China, which provided STR authentication reports. ACSL4^KO^ MEFs were kindly provided by Dr. Marcus Conrad, and more details can be obtained from [44]. A375, B16F10, MEF cells were cultured in DMEM (VivaCell, China), SK-MEL-2 and SK-MEL-28 cells were cultured in MEM (VivaCell, China) supplemented with 10% FBS at 37 °C in a humidified incubator. Cells were determined to be free from *Mycoplasma* contamination by Mycoplasma PCR Detection Kit (C0301S, Beyotime, China). Cells were cultured in 10-cm plates (Nest, 704001) and then transferred into a 96-well plate (Nest, 701001) to test cell viability, a 24-well plate (Nest, 702001) to test LPO and a 12-well plate (Nest, 712012) for observing cell morphology.

### Xenograft assay

All animal experiments were reviewed and approved by the Animal Ethical and Welfare Committee of Peking University (Approval No. LA2022689, Beijing, China) and were conducted in accordance with the Guidelines for Care and Use of Laboratory Animals published by the United States National Institutes of Health (Eighth edition; revised 2011). Male BALB/c nude mice (5–6 weeks), male BALB/c mice (5–6 weeks), and male C57BL/6 J mice (6–8 weeks) were purchased from the Department of Animal Science of Peking University Health Science Center and housed under specific pathogen-free conditions. Quantity-, sex-, age-, and weight-matched mice were randomly assigned to different experimental groups. For cell line-derived xenograft experiments, 100 µL of PBS containing 5 × 10^5^ B16F10 cells was injected subcutaneously into the right flank of the mice. Cisplatin was dissolved in PBS and intraperitoneally injected into mice at a dose of 1 mg/kg every 3 days. MβCD was dissolved in PBS and intraperitoneally injected into mice at a dose of 100 mg/kg every 3 days. For trigger LPO and the formation of lipid rafts in B16F10 cells in mice, ML210 was dissolved in 5% DMSO/10% ethanol/50% PEG 400/35% PBS to create a 25 mg/mL solution and subcutaneously injected into the mice around the tumor at a dose of 50 mg/kg every 2 days. Tumor growth was monitored using a calliper until the endpoint and calculated according to the equation volume = length × width^2 ^× 1/2. Mice were sacrificed when the tumor reached a volume of ~1500 mm^3^. Tumor rupture before the endpoint of the experiment were excluded from the analysis. Tumor tissues, heart, liver, spleen, lung, kidney, intestine, and peripheral blood were obtained for subsequent analysis. The investigators were not blinded to allocation during experiments and outcome assessment because of obvious identification.

### Immunostaining of tissue and cells

Mouse cell line-derived xenografts and organs were formalin-fixed and paraffin-embedded. Embedded tissues were sectioned at a thickness of 5 μm for haematoxylin-eosin (H&E) staining or immunofluorescence analysis. For immunofluorescence staining, after being deparaffinized and rehydrated, the sections were subjected to antigen retrieval to reveal epitopes by using sodium citrate antigen retrieval solution (C1032, solabio). Then, the sections were incubated with 5% bovine serum albumin (AP36L014, Life-iLab) for 1 h and incubated overnight at 4 °C with primary antibodies against 4-HNE (ab46544, goat, 1:100, Abcam) and HMGB1 (3935S, rabbit, 1:100, Cell Signaling Technology). The next day, the sections were washed and incubated with the appropriate secondary antibody Dylight 488 Conjugate anti-goat IgG (E032231, donkey, 1:200, EarthOx) or Dylight 594 Conjugate anti-rabbit IgG (E032421, donkey, 1:200, EarthOx) for 90 min at room temperature. Sections were mounted with anti-fade mounting medium and DAPI (S2100, Solarbio) and then images were acquired by the Vectra® Polaris™ Automated Quantitative Pathology Imaging System.

For immunostaining of cells, pretreated cells were fixed (4% PFA, 15 min at room temperature), permeated with 0.1% Triton X-100 for 3 min, incubated with 5% bovine serum albumin (AP36L014, Life-iLab) for 1 h, and incubated overnight at 4 °C with primary antibodies against HMGB1 (3935S, rabbit, 1:200, Cell Signaling Technology). The next day, cells were washed and incubated with Dylight 594 Conjugate anti-rabbit IgG at room temperature. Then, cells incubated with Alexa Fluor®488-Phalloidin (#8878, 1:20, Cell Signaling) for F-actin staining for 30 min and 5 min with DAPI for staining nuclei at room temperature. Images of cells were acquired by Nikon A1R SI confocal microscopy.

### Mass spectrometry-based untargeted lipidomics

Cells were seeded on 10-cm plates and treated as indicated for 24 h. A total of 10^7^ cells per sample were collected and centrifuged for 5 min at 100 g at 4 °C 3 times to remove the residual medium and PBS solution. Cells were mixed with 0.8 mL water from the Milli-Q® IQ 7015 water purification system, 1 mL chloroform, and 2 mL methanol and then shaken for 1 h at 4 °C. After adding 2 mL of chloroform and 1 mL of water to the samples, they were vortexed well and then centrifuged at 2000 × *g* for 10 min at 4 °C. The organic phase was collected, 1 mL of chloroform was added, and the mixture was vortexed and centrifuged to collect the organic phase. This process was repeated 3 times. Lipid extracts were concentrated by drying in a vacuum environment (to reduce lipid oxidation) and then reconstituted in 100 μL of chloroform before liquid chromatography and mass spectrophotometry (LC‒MS). LC‒MS was performed in a Q Exactive HF Orbitrap LC‒MS/MS System (Thermo Scientific). The LC part is an Ultimate 3000 UHPLC with an InertSustain C18 HP column (150 × 2.1 mm; 3 μm). The mobile phases were acetonitrile:water (60:40, v/v) + 10 mM ammonium formate and 0.1% formic acid (A) and acetonitrile:water (90:10, v/v) + 10 mM ammonium formate and 0.1% formic acid (B). The gradient elution time was 25 min with the following gradient elution programs of B: 0 min, 40% B; 2 min, 43% B; 2.1 min, 50% B; 12 min, 70% B; 18 min, 99% B; 20 min, 99% B; 20.5 min, 40% B; and 25 min, 40% B with a flow rate of 0.25 mL/min. MS-DIAL was used for baseline correction, retention correction, peak identification and alignment, and statistical analysis of the raw data.

### Transcriptomics

Total RNA was isolated from cells using TRIzol reagent (15596026, Invitrogen, USA) according to the manufacturer’s instructions, and genomic DNA was removed using DNase I (#2270 A, TaKara Bio, Shiga, Japan). RNA samples (OD260/280 = 1.8–2.2, OD260/230 ≥ 2.0, RIN ≥ 6.5, 28S:18S ≥ 1.0, >1 μg) were then subjected to library sequencing. Then, the messenger RNA was isolated according to the poly-A selection method by using oligo(dT) beads. Double-stranded cDNA was synthesized by using a SuperScript double-stranded cDNA synthesis kit (#11917010, Invitrogen) with random hexamer primers (#20022654, Illumina). A paired-end RNA-seq sequencing library was constructed with the Illumina HiSeq xten/NovaSeq 6000 sequencer (2 × 150 bp read length). Therawpaired end reads were trimmed and qualitycontrolled with default parameters by SeqPrep and Sickle. The clean reads were separately aligned to the reference genome with orientation mode by using HISAT2 software [63]. The mapped reads were assembled by StringTie according to a reference-based approach [64]. *P* adjusted value (Bonferroni-corrected) ≤ 0.05 and log2 (fold change) > 1 were considered statistically significant.

### Cell counting kit-8

Cells were seeded at a density of 3500 on 96-well plates overnight and then treated with different lethal compounds (RSL3 or ML210) and modulators (MβCD, LIP-1, ROSI, LDL, or ox-LDL) for 24 h. Viable cells on 96-well plates were measured using a Cell Counting Kit-8 kit (AC11L054, Life-iLab). Briefly, 10 μL CCK-8 reagent was added to 100 μL medium per well for 1 h at 37 °C in an incubator. The absorbance at a wavelength of 450 nm was measured by a Thermo Scientific Multiskan SkyHigh microplate reader.

### Lipid peroxidation assay

LPO was measured by C11-BODIPY 581/591 (D3861, Invitrogen). Briefly, cells were seeded on 24-well plates and incubated overnight in an incubator. After treatment with different compounds for the indicated time, the culture medium was discarded, and the cells were washed twice with PBS. Cells were then incubated with the kit reagent (5 μM in medium without FBS) for 30 min at 37 °C and then harvested and resuspended in 200 μL of medium. LPO levels were measured using flow cytometry (FITC channel, CytoFLEX). Three replicate samples in each group were analyzed, and 1 × 10^4^ cells per sample were analyzed.

### MDA measurement

For cells, 2 ×${10}^{6}$ cells were harvested and washed twice with cold PBS. For xenografts, ~25 mg tumor tissue was used. Cells or tissue lysed with RIPA buffer (AP01L013, Life-iLab) supplemented with cocktail proteinase inhibitor and centrifuged at 15,000 × *g* for 15 min at 4 °C. For measuring the MDA content following the manufacturer’s instructions (S0131S, Beyotime Biotechnology), the collected supernatants (100 μL) and 3 μL antioxidants were added to 200 μL of 0.37% TBA reagent (diluted with 5% trichloroacetic acid), and the mixture was incubated at 95 °C for 60 min. Meanwhile, the protein content of the collected supernatants was determined by BCA assay and equilibrated. When the samples were at room temperature, the MDA-TBA adduct fluorescence intensity (λex = 532/λem = 553 nm) for cells or OD 532 nm for tissue was measured.

### Western blot analysis

Total protein was extracted from treated cells or tumor tissues in RIPA lysis buffer containing protease-phosphatase cocktail inhibitor mix. The protein concentration was measured by a BCA Protein Assay Kit and then separated by SDS-PAGE in 10% polyacrylamide gels (M00664, GenScript) and electrically transferred to a PVDF membrane (Bio-Rad). 5% skim milk was used to block nonspecific antigens and incubated overnight at 4 °C with primary antibodies as follows: Caspase 3 (ab32351, 1:1000, Abcam), Cleaved Caspase 3 (9664T, 1:1000, Cell Signaling Technology), GSDME (ab215191, 1:1000, Abcam), 4-HNE (ab46544, 1:1000, Abcam), FLOT-1 (ab133497, 1:1000, Abcam), ACSL4 (ab155282, 1:1000, Abcam), GAPDH (60004-1-Ig, 1:5000, Proteintech), and β-actin (bsm-33036M, 1:500, Bioss). Full and uncropped western blots, uploaded as ‘Supplementary materials-Western blot original images’.

### Enzymatic tumor disaggregation

The samples were washed twice with PBS and rinsed with serum-free 1640 medium (#31870082, Gibco) under sterile conditions. The samples were then split into parts (~0.5 × 0.5 × 0.5 mm) with scissors and covered with 10% (v/v) FBS 1640 medium containing 1 mg/mL type I collagenase (#17100017, Gibco) at 37 °C for disaggregation. Tumor particles were pipetted vigorously to enhance disaggregation every 45 min. After ~3 h, the samples were filtered through a 70 µm Cell Strainer (#352350, Falcon), and the suspensions were centrifuged for 5 min at 300 g at room temperature. The collected cells were then cultured in 10-cm plates for further experiments.

### Lipid raft staining

Lipid raft labeling was conducted using the Vybrant™ Alexa Fluor™ 555 Lipid Raft labeling kit according to the manufacturer’s protocol (V34404, Invitrogen). Briefly, cells were washed with cold PBS, labeled with Alexa-Fluor-555-conjugated CTx-B at 4 °C for 10 min, and then cross-linked with anti-Ctx-B antibody for 15 min. Thereafter, the cells were fixed using 4% paraformaldehyde at room temperature for 15 min. Nuclei of cells were stained with DAPI dihydrochloride (C0065, solabio). Images were captured through Nikon A1R SI confocal microscopy.

### Overexpressed gasdermin-N domains

Gasdermins A-E share a highly conserved N-domain. The gasdermin-N domains, but not the full-length proteins, induced extensive pyroptosis [35, 46]. We overexpressed the GSDMD-N domain to induce pyroptosis in vitro. The sequence of FLAG-GSDMD-NT (80951) was obtained from Addgene and generated by GENEWIZ [65]. DH5 Competent Cells (TSC-C14, TSINGKE, China) are used to amplify, and a single colony was taken and seeded into 10 mL of liquid LB medium with ampicillin (B540112-0100, Sango Biotech). After incubation at 37 °C and 220 rpm overnight, the liquid was centrifuged at 8000 × *g* for 10 min. The supernatant was discarded, and the plasmid extraction assay was conducted using an EndoFree Plasmid Midi Kit (CoWin Biosciences) according to the manufacturer’s instructions. Finally, the plasmid DNA was quantified with Nanodrop One followed by storage at −20 °C until further use. Transient transfection of cells was performed using Lipofectamine 3000 (L3000008, Invitrogen) according to the manufacturer’s instructions.

### LDH assay

Cell culture supernatants were collected and centrifuged at 500 × *g* for 5 min to remove cellular debris. The LDH assay was performed with the CytoTox 96 Non-Radioactive Cytotoxicity Assay (G1780, Promega) according to the manufacturer’s instructions. Data were normalized to the OD value obtained in negative control wells treated with Triton X-100 at 100%.

### Measurement of released ATP

Cells were treated with the indicated compounds at the appropriate times. Then, the supernatants were collected and centrifuged at 15,000 rpm at 4 °C for 3 min. The supernatants were used immediately for ATP. ATP analysis was conducted using a CellTiter-Glo luminescent cell viability assay kit (Promega, G7571) according to the manufacturer’s instructions. The luminescence was measured on a BioTek Synergy Neo2.

### Cell membrane integrity measurement

Cells were seeded in 24-well plates and treated with RSL3, LDL or ox-LDL for the indicated times. Then harvested cells and treated with 0.1% Triton X-100 for indicated times. After that, the treated cells stained with propidium iodide (PI) at a final concentration of 4 μg/mL. Flow cytometry (CytoFLEX) was used to measure cell membrane integrity. Three replicate samples in each group were measured, and a total of 1 × 10^4^ cells in each sample were analyzed.

### Analysis of BMDC maturation

Over 7 days, BMDCs were differentiated from the femurs and tibias of C57BL/6J mice using RPMI culture medium (GIBCO) supplemented with 10% FBS, 1% L-glutamine, interleukin 4 (10 ng/mL), 2-mercaptoethanol (50 μM), pyruvate (1 mM) and mouse granulocyte macrophage colony stimulating factor (20 ng/ml). The culture medium was refreshed on day 2 and day 4. Then, the obtained BMDCs were co-incubated with dying B16F10 cells at ratios of 1:10 for 18 h. In addition, BMDCs were stimulated with 100 ng/mL *E. coli* lipopolysaccharide (LPS) as a quality control. The cells were then centrifuged at 400 × *g* for 6 min at 4 °C, washed with PBS, and stained with anti-CD11c APC (#117309, BioLegend), anti-CD86 APC-Cy7 (#105029, BioLegend), anti-MHC-II PE-Cy7 (#107629, BioLegend), and mouse Fc-block (#156603, BioLegend) antibodies.

### Lipid raft assay

Then, the cells were harvested in 0.5 ml of the initial lysis buffer (PBS containing 0.05% Triton X‐100, 1 mM phenylmethylsulfonyl fluoride, and 1 × protease inhibitor cocktail), mixed by vortexing and incubated on ice for 10 min. The samples were centrifuged at 20,000 × *g* for 10 min at 4 °C, and the supernatants were discarded. Then, the sediments were resuspended in 50 μl of B buffer from the UltraRIPA kit for Lipid Raft (BioDynamics Laboratory Inc., Tokyo, Japan), rigorously vortexed, incubated at room temperature for 5 min, and centrifuged at 20,000 × *g* for 10 min. Finally, the supernatants containing lipid raft protein were collected, and protein concentrations were measured by a BCA Protein Assay Kit.

### Cell morphology

Cells were seeded in 12-well plates and treated with the indicated chemicals, and then, micrographs of cell cultures were obtained under phase contrast illumination using a 20X objective (Leica) prior to cell lysis.

### Mouse spleen and tumor-infiltrating T lymphocyte extraction and cytokine detection

To obtain spleen T cells, the mouse spleen was washed with PBS and ground on a 200-mesh filter to make a single-cell suspension. The red blood cells in suspension were lysed with red blood cell lysis buffer (Solarbio) according to the manufacturer’s protocol. To obtain tumor-infiltrating T cells, tumor tissues were minced into small pieces in 1640 medium containing 2% FBS, 0.5 mg/ml DNase I (Sigma‒Aldrich), and 0.5 mg/mL collagenase Type I (Sigma‒Aldrich) and then incubated for digestion for 3 h at 37 °C, followed by filtration with 70 µm Cell Strainers (#352350, Falcon). T cells were enriched by a mouse tumor infiltrating tissue lymphocyte isolation fluid kit (P9000, Solarbio). For cytokine staining, T cells were incubated in culture medium containing PMA (5 ng/mL, TargetMol), ionomycin (500 ng/ml, TargetMol) and brefeldin A (1:1000, TargetMol) at 37 °C for 4 h. To reduce the interference of dead cells on subsequent detection, cells were then resuspended in PBS containing 1 μL of Fixable Viability Dye eFlour506 (Thermo Fisher Scientific) for 20 min. Thereafter, the cells were washed with PBS and collected for subsequent surface antigens and intracellular cytokine staining. Briefly, anti-CD45 (#103115, Biolegend), anti-CD3 (#100202, Biolegend), anti-CD4 (#100513, Biolegend), and anti-CD8a (#100722, Biolegend) antibodies were added for 20 min for surface staining. The cells were then washed and fixed in 4% formaldehyde (Sigma Aldrich). After being washed with PBS containing 2% FBS, the cells were stained with anti-TNFα (#506307, Biolegend) and anti-IFNγ (#505808, Biolegend) for 30 min. All samples were read on a flow cytometer (CytoFLEX).

### Mouse spleen DC cell extraction and maturity level test

Cells from spleen were obtained as described above. Collected cells were then subjected to LIVE/DEAD staining with Fixable Viability Dye eFlour506 (Thermo Fisher Scientific) for 20 min, followed by surface antigen staining with anti-CD11c (#117309, Biolegend) and anti-MHC II (#107629, Biolegend) for 20 min. All samples were read on a flow cytometer (CytoFLEX).

### Statistical analysis

All data are represented as the mean ± SEM. Statistical analyses were performed using GraphPad Prism 9.3.0 software. Differences between two groups were analyzed by Student’s *t* test, whereas ANOVA is used to compare the means among three or more groups. The results of cell culture experiments were obtained from at least three independent replicates. Volumes or weights from at least five tumors in each group were plotted. Adjustments for multiple comparisons were not made in this study. A two-tailed *p* < 0.05 was considered statistically significant. *P < 0.05; **P < 0.01; ***P < 0.001; ****P < 0.0001; NS, nonsignificant. No statistical methods were used to predetermine the sample size. The investigators were not blinded to allocation during experiments and outcome assessment because of obvious identification.

## Conclusions

Disruption of lipid rafts by depleting membrane cholesterol significantly enhances platinum-based drug-triggered immunogenic ferroptosis and pyroptosis, while thereafter ameliorating the immunosuppressive microenvironment through the activation of dendritic cells and cytotoxic T cells. This approach of lipid raft disruption represents a promising novel strategy for alleviating drug-resistance and complementing ICB therapy in melanoma.

## Supplementary information

Supporting information

Original western blots

## Data Availability

All data that support the conclusions in this manuscript are available from the corresponding author upon reasonable request.
