# Supplementary material for: ACSL4-mediated lipid rafts prevent membrane rupture and inhibit immunogenic cell death in melanoma
Source: Cell Death Dis. 2024 Sep 29;15(9):695. doi: 10.1038/s41419-024-07098-3 (PMC11439949; doi:10.1038/s41419-024-07098-3)
Supplement: Supplementary file 1 — Supporting information [file 41419_2024_7098_MOESM1_ESM.docx]

**Supplementary Information for**

**ACSL4-mediated Lipid Rafts Prevent Membrane Rupture and Inhibit Immunogenic Cell Death in Melanoma**

Xi Zhao^1,2 #^, Zenglu Zhao^1,2 #^, Bingru Li^1,2^, Shuyu Huan^1,2^, Zixi Li^1,2^, Jianlan Xie^3^, Guoquan Liu^1,2,4 *^

^1^ State Key Laboratory of Natural and Biomimetic Drugs, School of Pharmaceutical Sciences, Peking University, Beijing, China

^2^ Department of Pharmaceutical Analysis, School of Pharmaceutical Sciences, Peking University, Beijing, China

^3^ Department of Pathology, Beijing Friendship Hospital, Capital Medical University, Beijing, China

^4^ Department of Biomedical Engineering, Institute of Advanced Clinical Medicine, Peking University, Beijing 100191, China

^#^ These authors contributed equally

* Corresponding author, email: guoquanliu@bjmu.edu.cn

**Supplementary Figures**


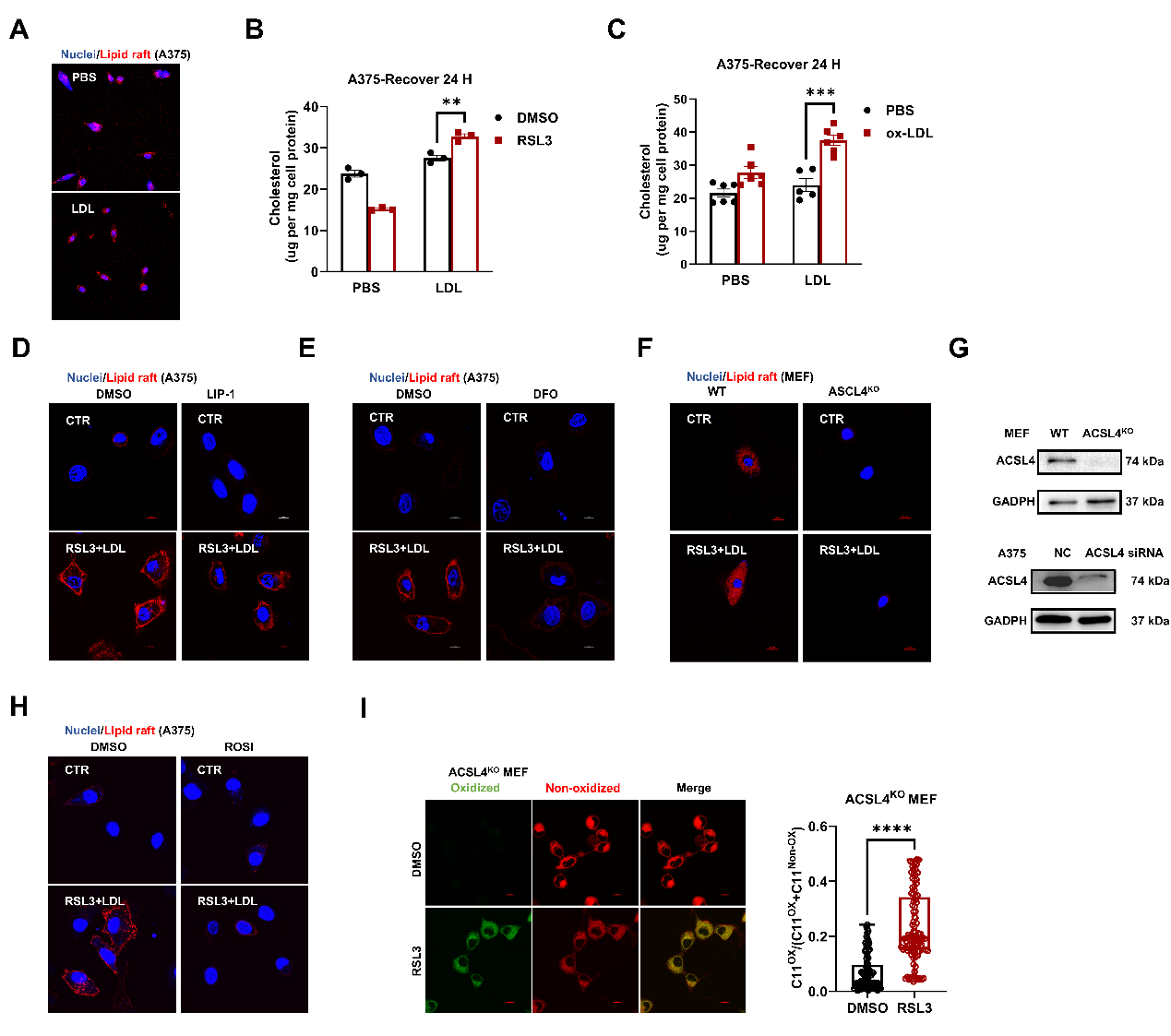


**Figure S1. Confocal images verified that LPO participation in the formation of lipid rafts is ACSL4 dependent.** **A, D-F, H.** Effect of LDL (**A**, 40 μg/mL) or LIP-1 (2 μM, **D**) or DFO (50 μM, **E**) or ACSL4^KO^ (**F**) or rosiglitazone (ROSI, 50 μM, **H**) on lipid rafts in A375 cells (**A, D-E, H**) or MEFs (**F**). **B-C.** The content of cholesterol in A375 cells treated as indicated for 24 h after withdraw treatment agents and culture for another 24 h. **G.** Validation of ACSL4 knockout by Western blotting. **I.** Images and quantification of C11-BODIPY oxidation ratios in RSL3 (0.5 μM)-treated ACSL4^KO^ MEFs. Each data point represents an individual cell, with approximately 50-100 cells randomly counted for statistical analysis. Statistical significance was assessed using an unpaired two-tailed t test (**I**).


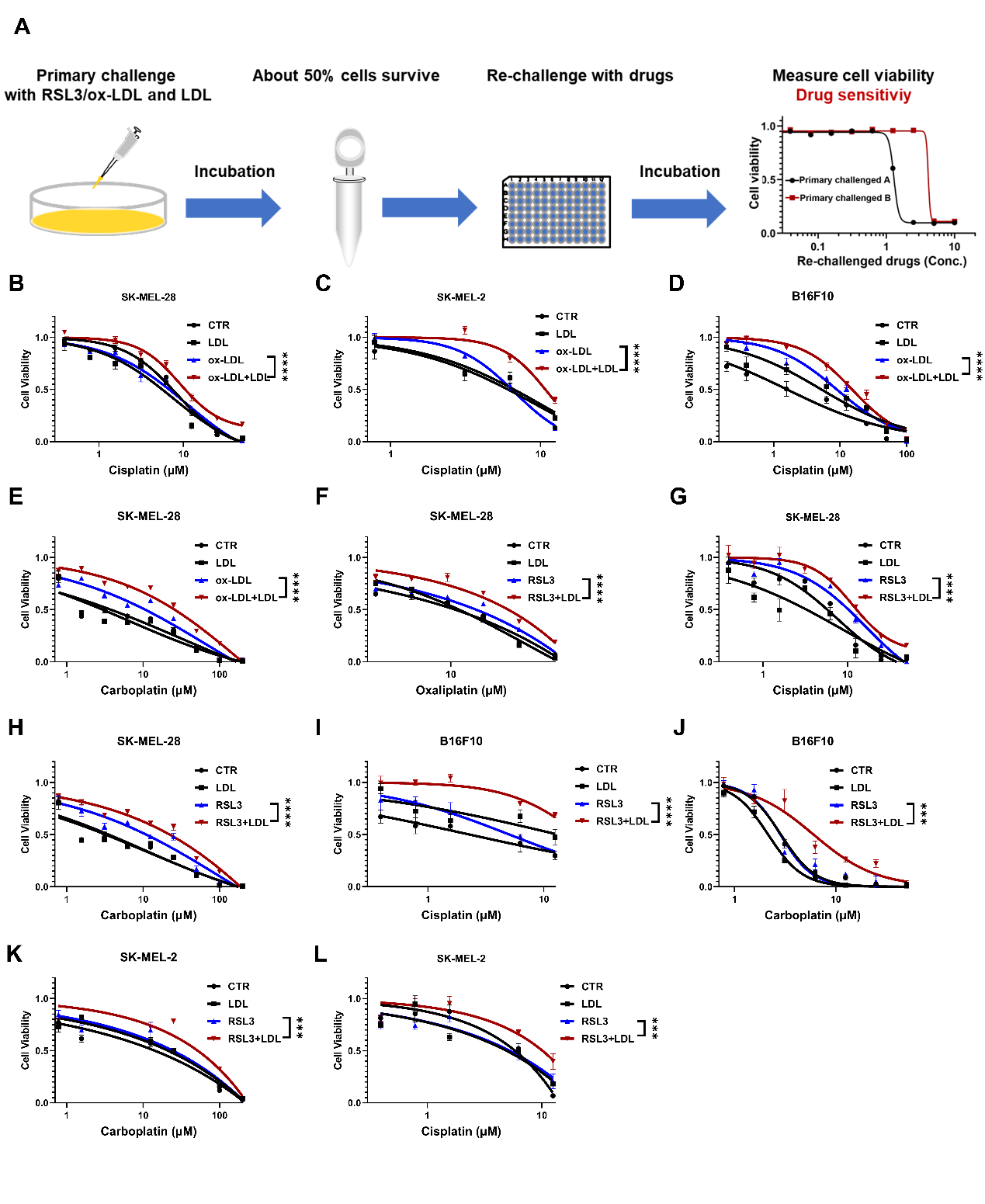


**Figure S2. Effect of the modulators on the sensitivity to platinum-based drugs in multiple cancer cells. A.** Scheme of the modulatory profiling experiment. **B-L.** Changes in melanoma cell line sensitivity to platinum-based drugs after RSL3 or RSL3 plus LDL or ox-LDL plus LDL treatment for 24 h. LDL is 40 μg/mL, and ox-LDL is 20 μg/mL. The results are derived from 3 independent replicates. Statistical significance was assessed using two-way ANOVA.


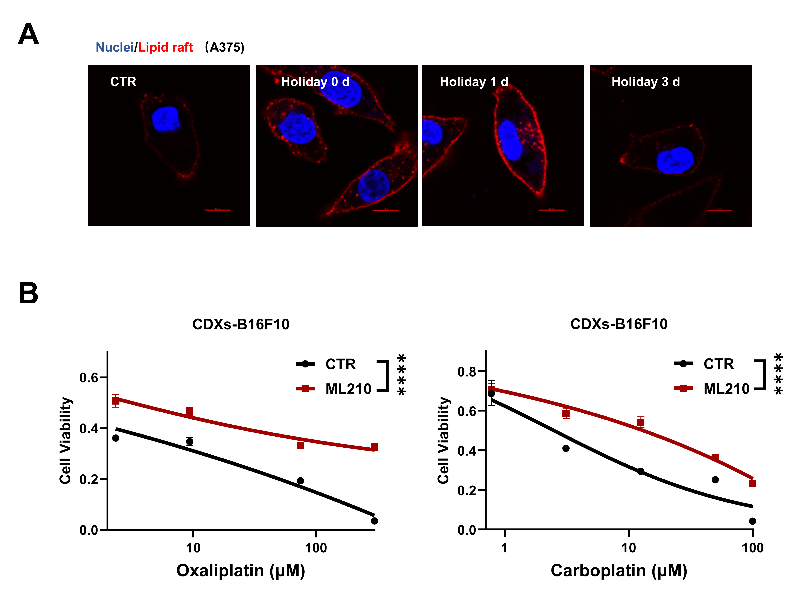


**Figure S3.** **Decreased sensitivity in B16F10 cells derived from ML210-treated xenografts.** **A.** Time evolution of lipid rafts in A375 cells after LDL and RSL3 treatment to acquire lipid rafts. **B.** The dose-dependent cytotoxicity of oxaliplatin and carboplatin on vehicle-treated xenograft-derived B16F10 cells or ML210-treated cells. The results are derived from 3 independent replicates. Statistical significance was assessed using two-way ANOVA.


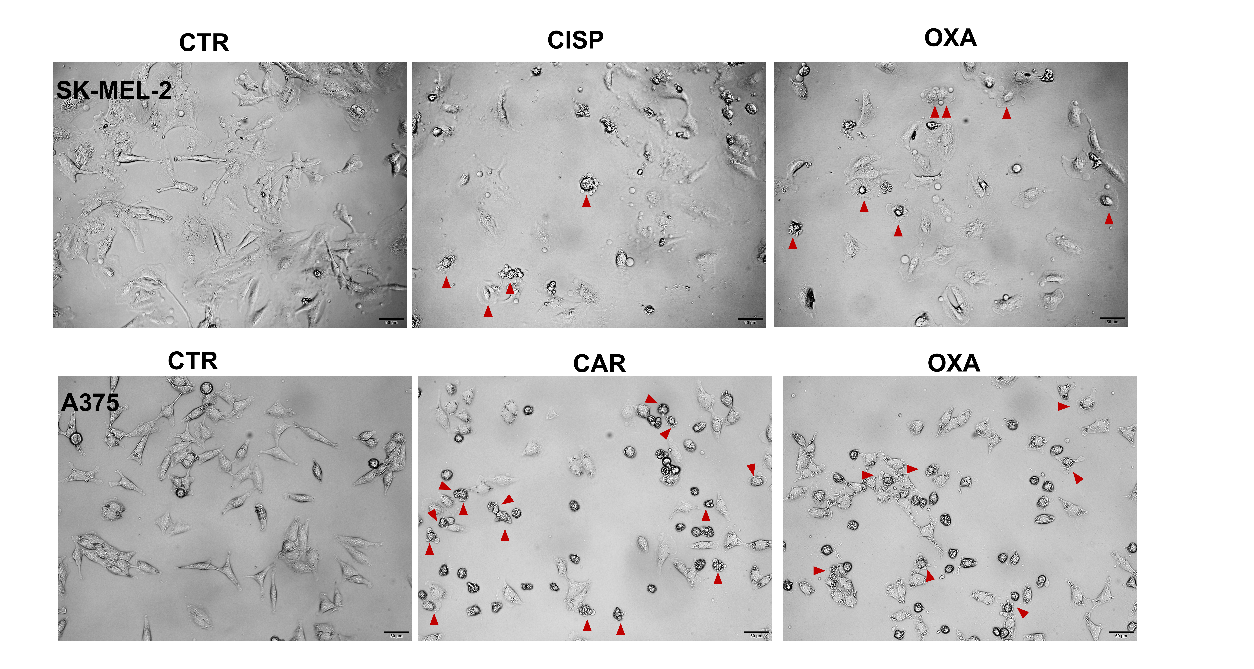


**Figure S4.** **Representative images of inverted phase-contrast microscopy of pyrolytic cells.** The red triangle indicates that the cell is undergoing pyroptosis. Scale bars=50 μm.


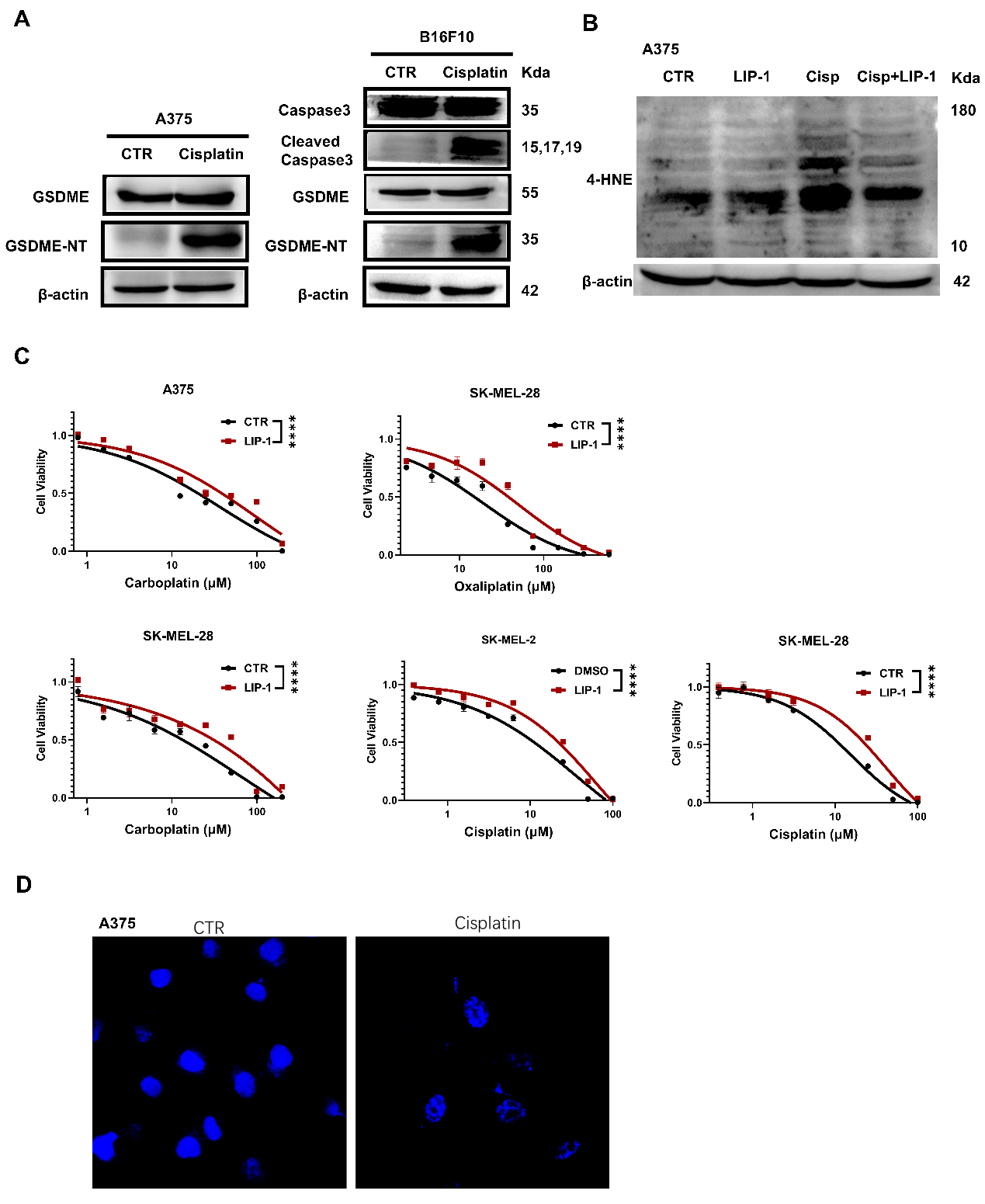


**Figure S5.** **Platinum-based drugs induced multiple forms of death. A.** The activation of GSDME and caspase 3 in A375 and B16F10 measuring by Western blotting. **B.** LIP-1 (2 μM) decrease 4-HNE-modified proteins triggered by cisplatin in A375 cells. **C.** LIP-1 desensitizes melanoma cell lines cells to platinum-based drugs. The results are derived from 3 independent replicates. **D.** Apoptotic cell death was demonstrated by the characteristic shrinkage of cellular nuclei. Blue indicates DAPI-stained nuclei. Statistical significance was assessed using two-way ANOVA (**C**).


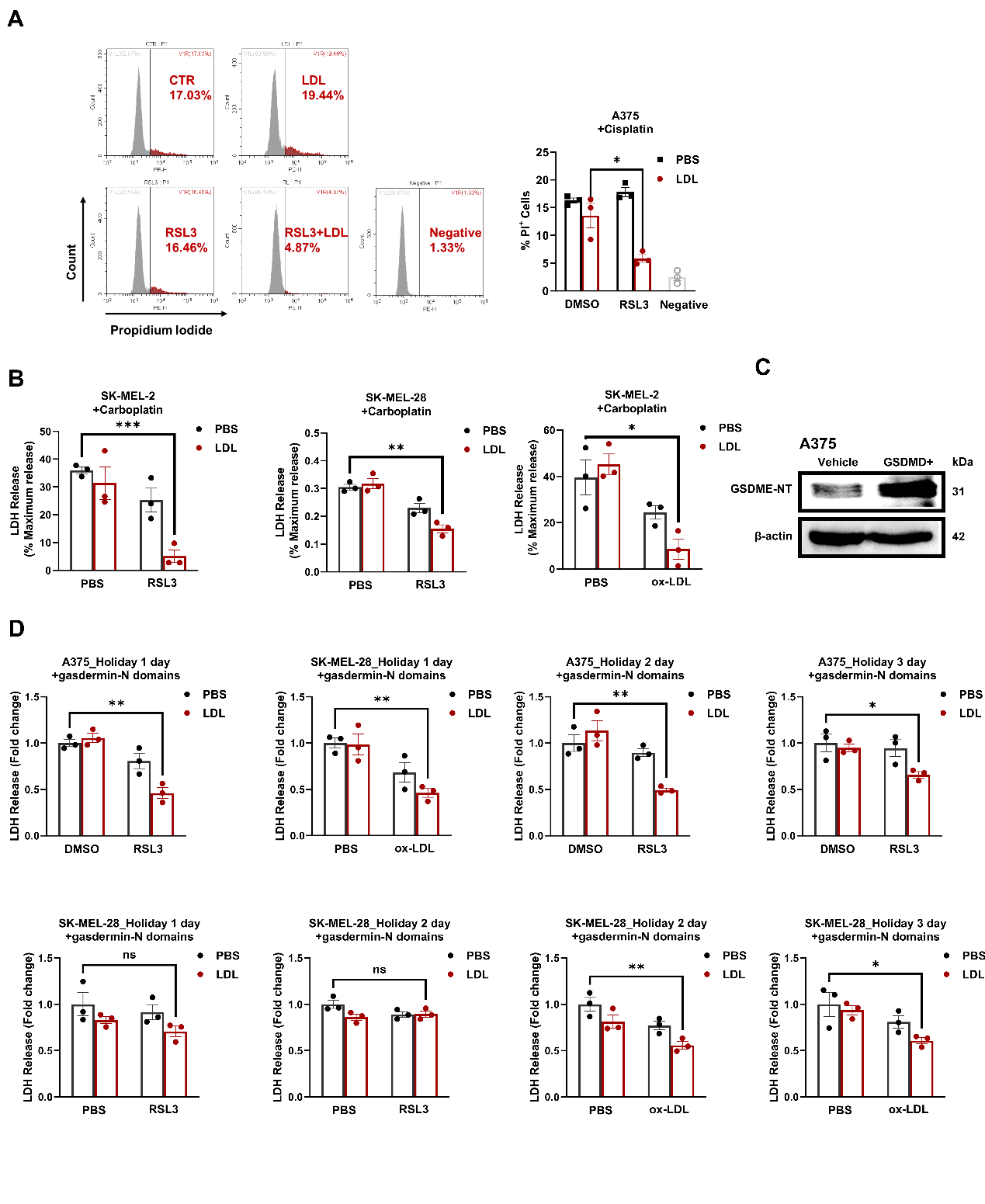


**Figure S6.** **Lipid raft formation reduced the sensitivity of melanoma cells to ferroptosis or pyroptosis. A.** Changes in A375 cell sensitivity to cisplatin (25 μM) assayed by PI staining after the indicated pretreatment (LDL 40 μg/mL, RSL3 2 μM, treated for 24 h). Each data point represents an independent repeat. **B.** Changes LDH release induced by cisplatin or carboplatin after the indicated pretreatment in melanoma cell lines. Each data point represents an independent repeat. **C.** Validation of gasdermin-N domains overexpression by Western blotting. **D.** Time evolution of melanoma cell lines sensitivity to GSDME-NT overexpression-induced pyroptosis after the indicated pretreatment. Each data point represents an independent repeat. Statistical significance was assessed using an unpaired two-tailed t test.


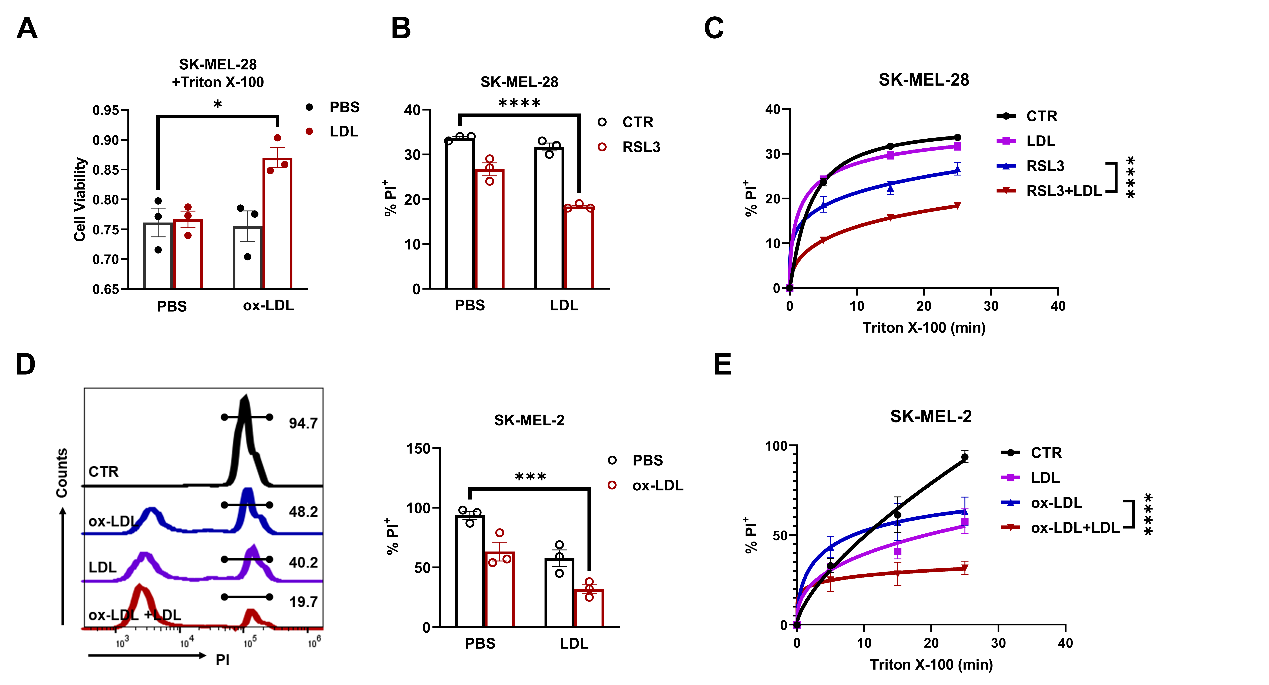


**Figure S7.** **Lipid raft reduced membrane pore-forming. A.** Cell viability of SK-MEL-28 cells with indicated pretreatment following Triton X-100 treatment. **C-E.** PI intensity and positive rate of SK-MEL-28 cells (**B-C**) or SK-MEL-2 cells (**D-E**) with indicated pretreatment following Triton X-100 treatment examined by flow cytometry. A-B, D. Each data point represents an independent repeat. C, E. The results are derived from 3 independent replicates. Statistical significance was assessed using two-way ANOVA (**C, E**) or an unpaired two-tailed t test (**A-B, D**).


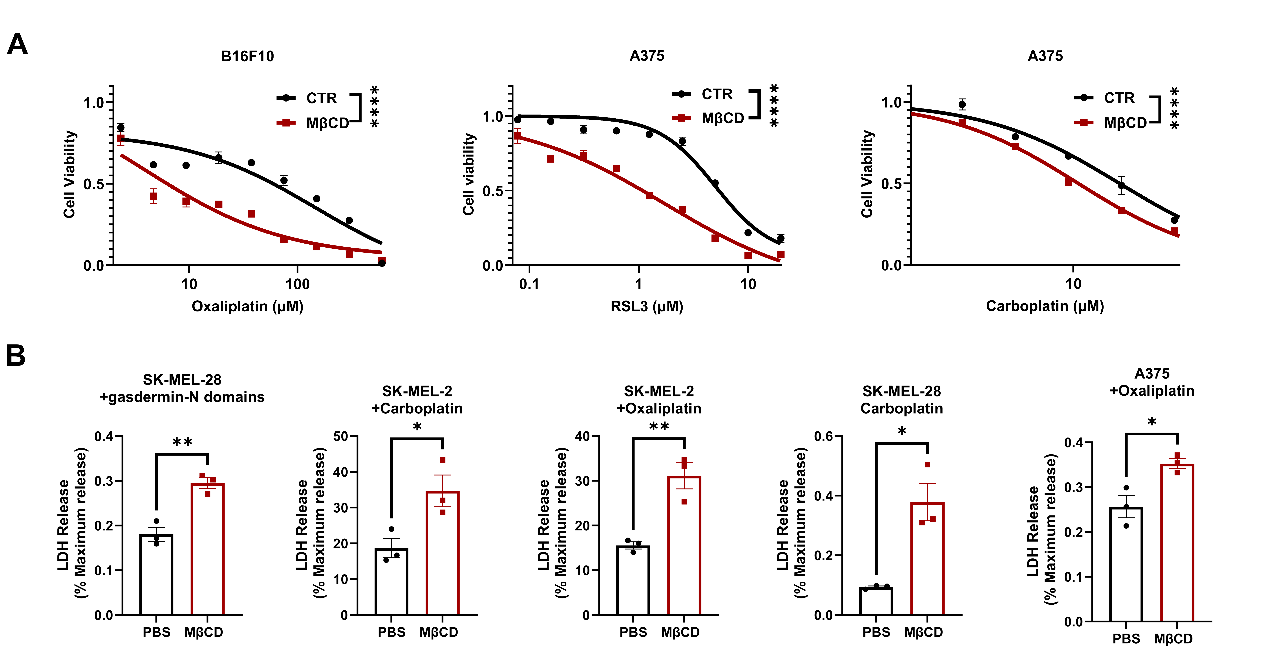


**Figure S8.** **MβCD disrupting lipid rafts enhanced the efficacy of platinum-based drugs in melanoma. A-B.** Effect of MβCD on melanoma cell line sensitivity to platinum-based drugs and gasdermin-N domain overexpression-induced pyroptosis. **A.** The results are derived from 3 independent replicates. **B.** Each data point represents an independent repeat. Statistical significance was assessed using two-way ANOVA (**A**) or an unpaired two-tailed t test (**B**).


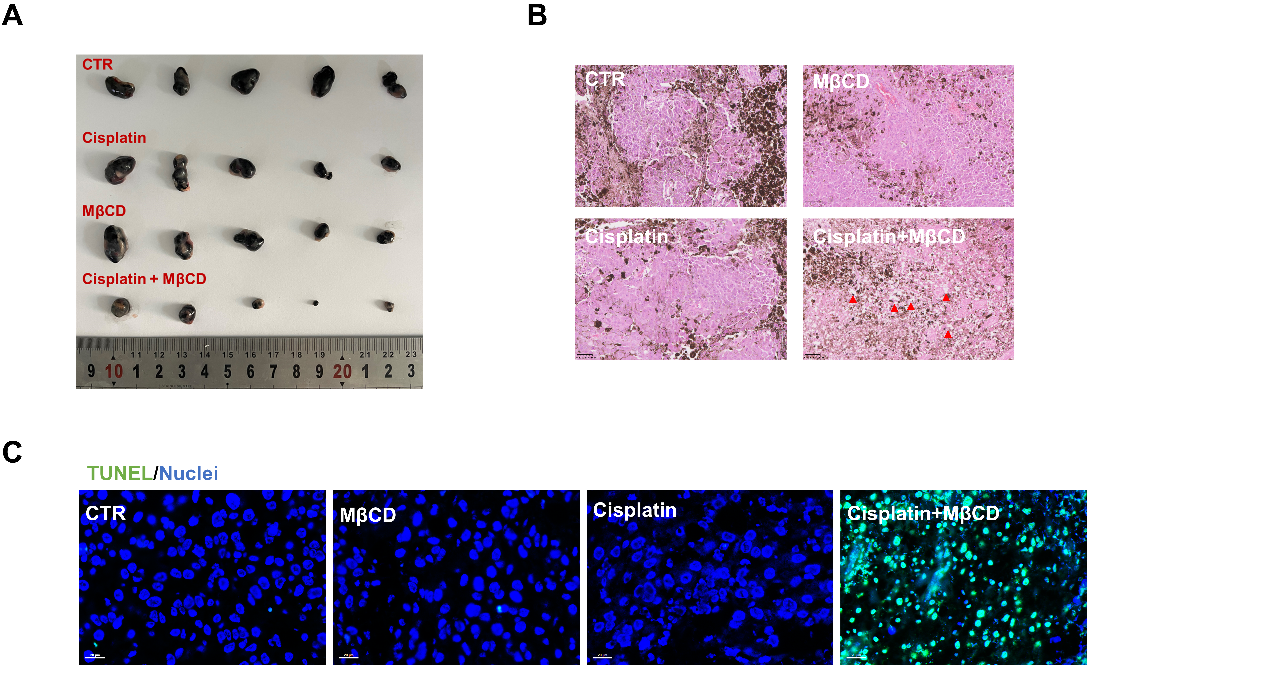


**Figure S9.** **MβCD disrupting lipid rafts promoted cisplatin antitumour activity. A.** Images and weight of xenografts obtained at the end of indicated treatments. **B.** Histological examination of tumors by H&E staining. Red triangles indicate vacuoles. Scale bars = 50 μm. **C.** Fluorescence staining of TUNEL (green) in paraffin-embedded xenografts at the end of the indicated treatment. Blue indicates DAPI-stained nuclei. Scale bars = 20 μm.


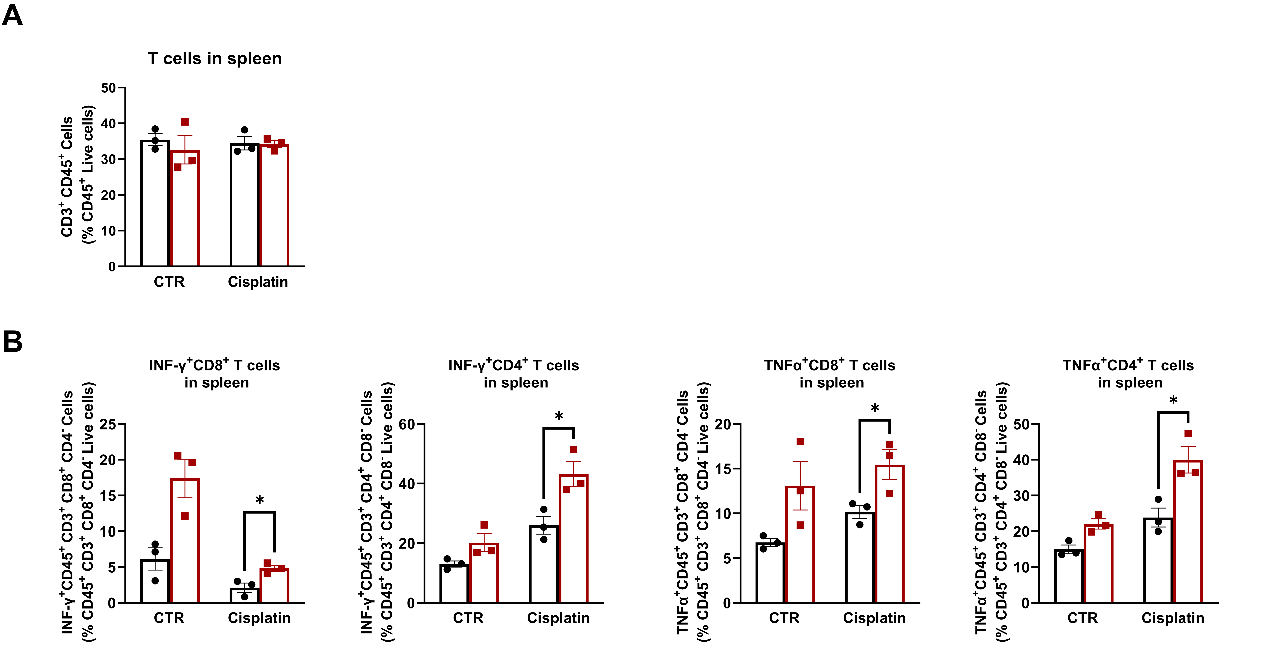


**Figure S10.** **IFN-γ and TNFα were obviously upregulated in spleen of MβCD and cisplatin treated mice. A.** Percentages of CD3^+^ T cells in CD45^+^ cells of B16F10 tumors. **B.** Percentages of IFNγ^+^ CD8^+^, IFNγ^+^ CD4^+^, TNFα^+^ CD8^+^, TNFα^+^ CD4^+^ T cells of B16F10 tumors. **A-B.** Each data point represents a tumor sample, 3 per group used for flow cytometric analysis. Statistical significance was assessed using an unpaired two-tailed t test (**A-B**).


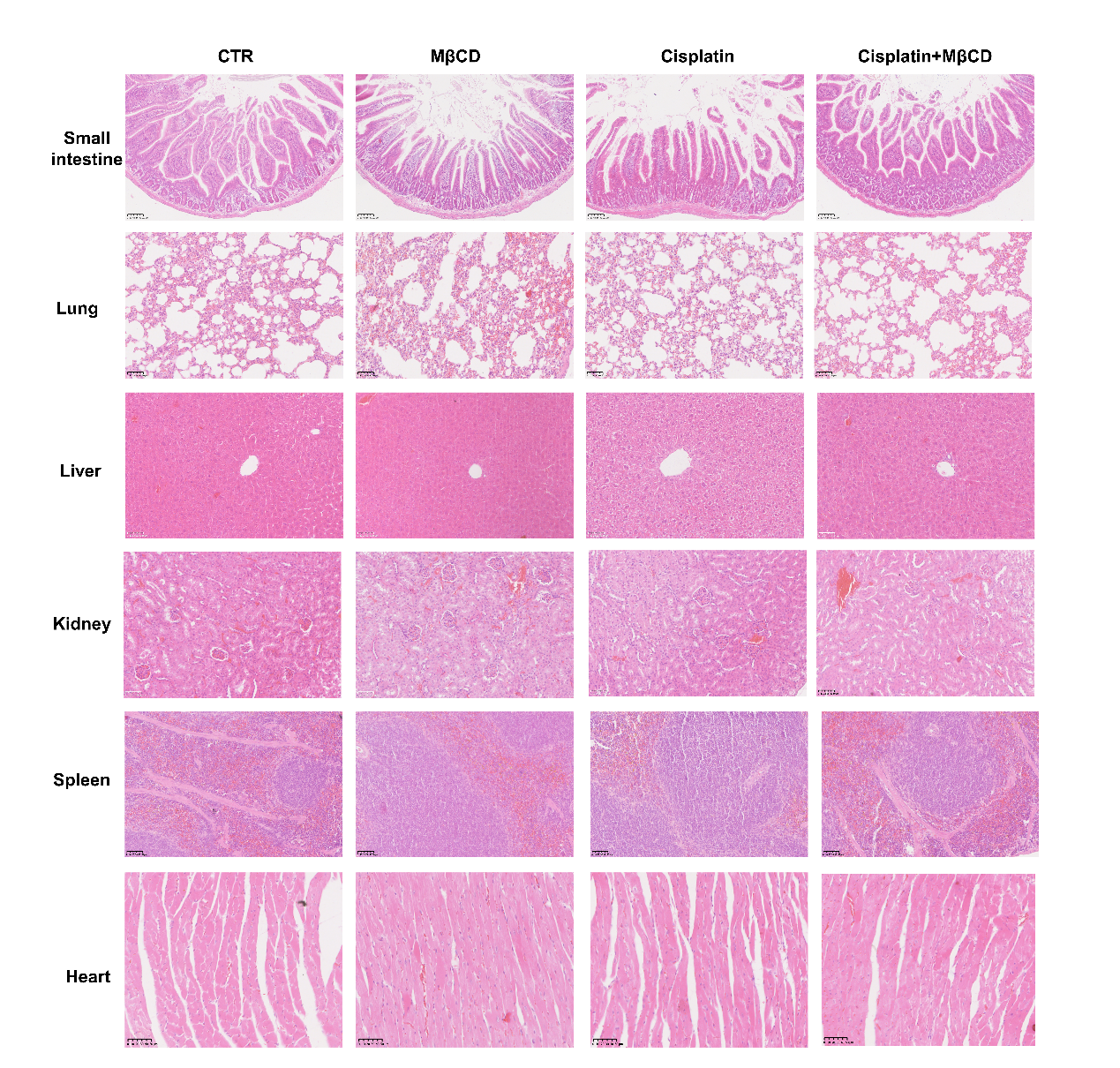


**Figure S11. Histological examination of organs by H&E staining.**


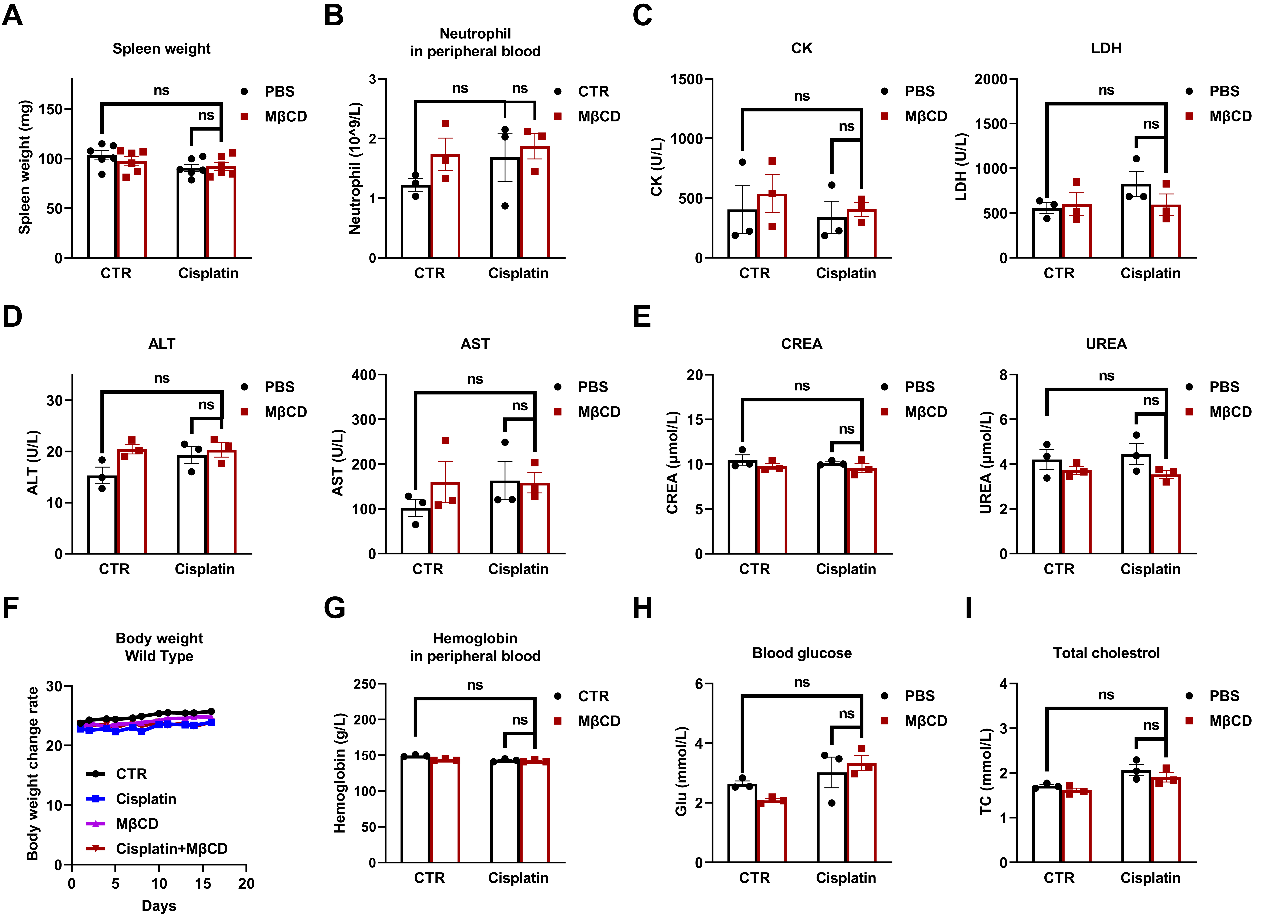


**Figure S12. The hematological analysis in vivo. A.** Statistics for spleen weight **o**f BALB/c mice with the indicated treatments. Each data point represents a spleen sample, each group consisted of 6. **B.** Total neutrophil number in peripheral blood of mice with indicated treatment. **C-E.** Blood biochemistry analysis of mice, including CK, LDH, ALT, AST, CREA and UREA. **F.** Weights of BALB/c nude mice for B16F10 xenografts with different treatments at different time points (days). **G-I.** Statistical analysis of nutritional indicators in peripheral blood, including hemoglobin, glucose and total cholesterol. **B-E, G-I.** Each data point represents a blood sample, 3 per group used for analysis. Statistical significance was assessed using an unpaired two-tailed t test.


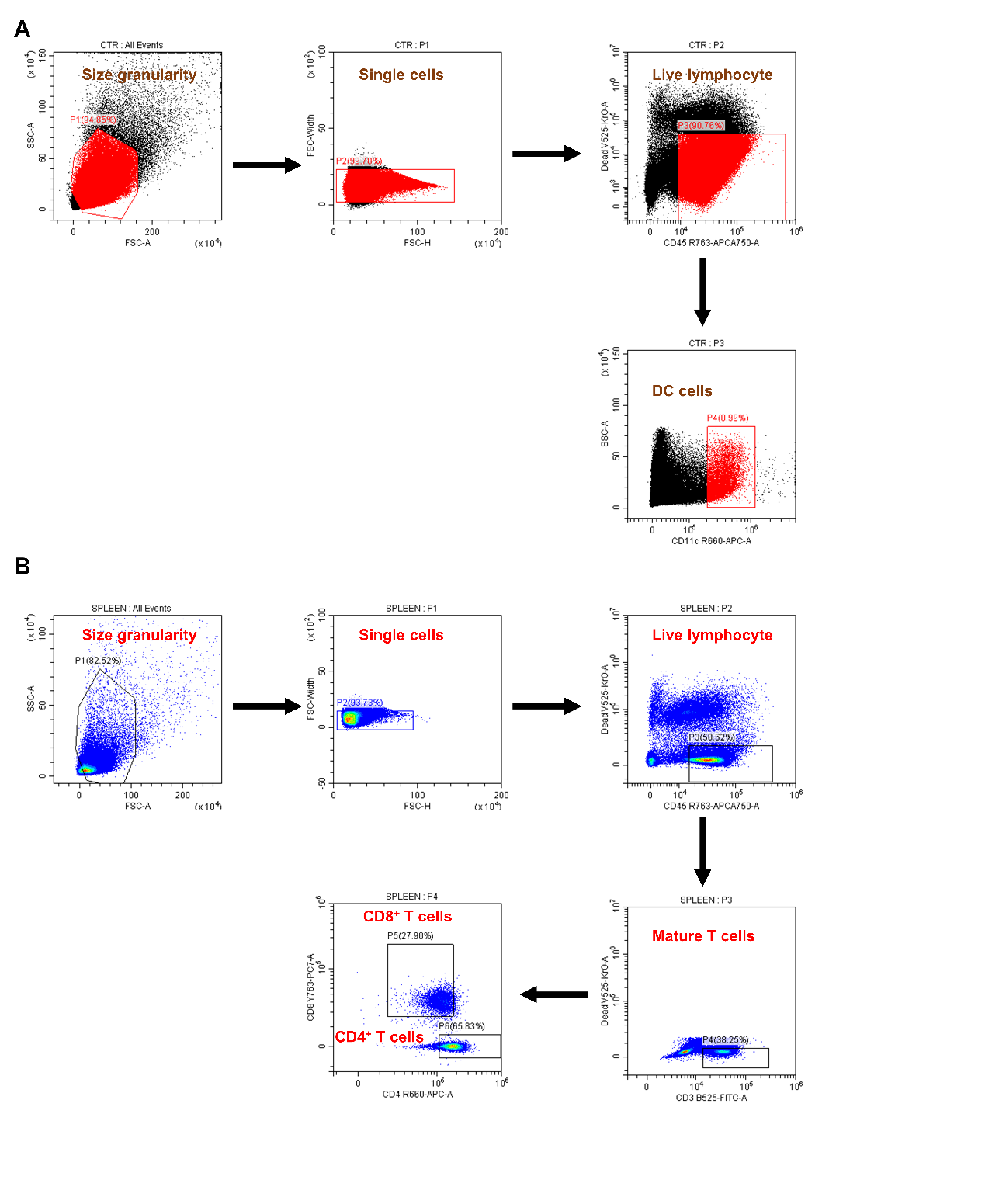


**Figure S13.** **Gating strategy for the flow cytometric immunophenotyping of lymphocytic cell subtypes.** **A.** Identification of DC cells. **B.** CD8^+^ T cells and CD4^+^ T cells.
