# Supplementary material for: ACSL4-mediated lipid rafts prevent membrane rupture and inhibit immunogenic cell death in melanoma
Source: Cell Death Dis. 2024 Sep 29;15(9):695. doi: 10.1038/s41419-024-07098-3 (PMC11439949; doi:10.1038/s41419-024-07098-3)
Supplement: Supplementary file 2 — Original western blots [file 41419_2024_7098_MOESM2_ESM.pptx]

## Slide 1
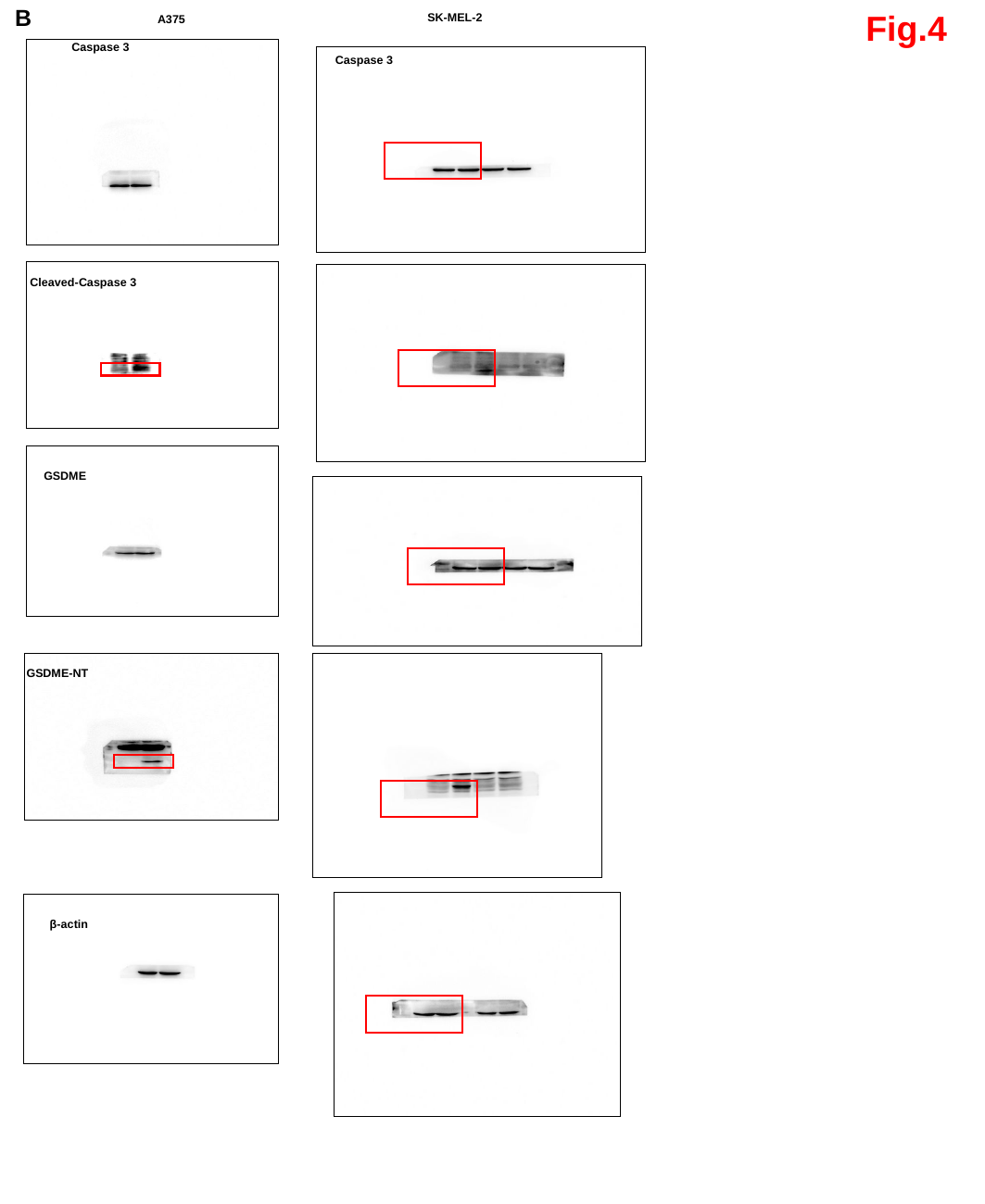

Fig.4
B
SK-MEL-2
A375
Caspase 3
Caspase 3
Cleaved-Caspase 3
GSDME
GSDME-NT
β-actin

## Slide 2
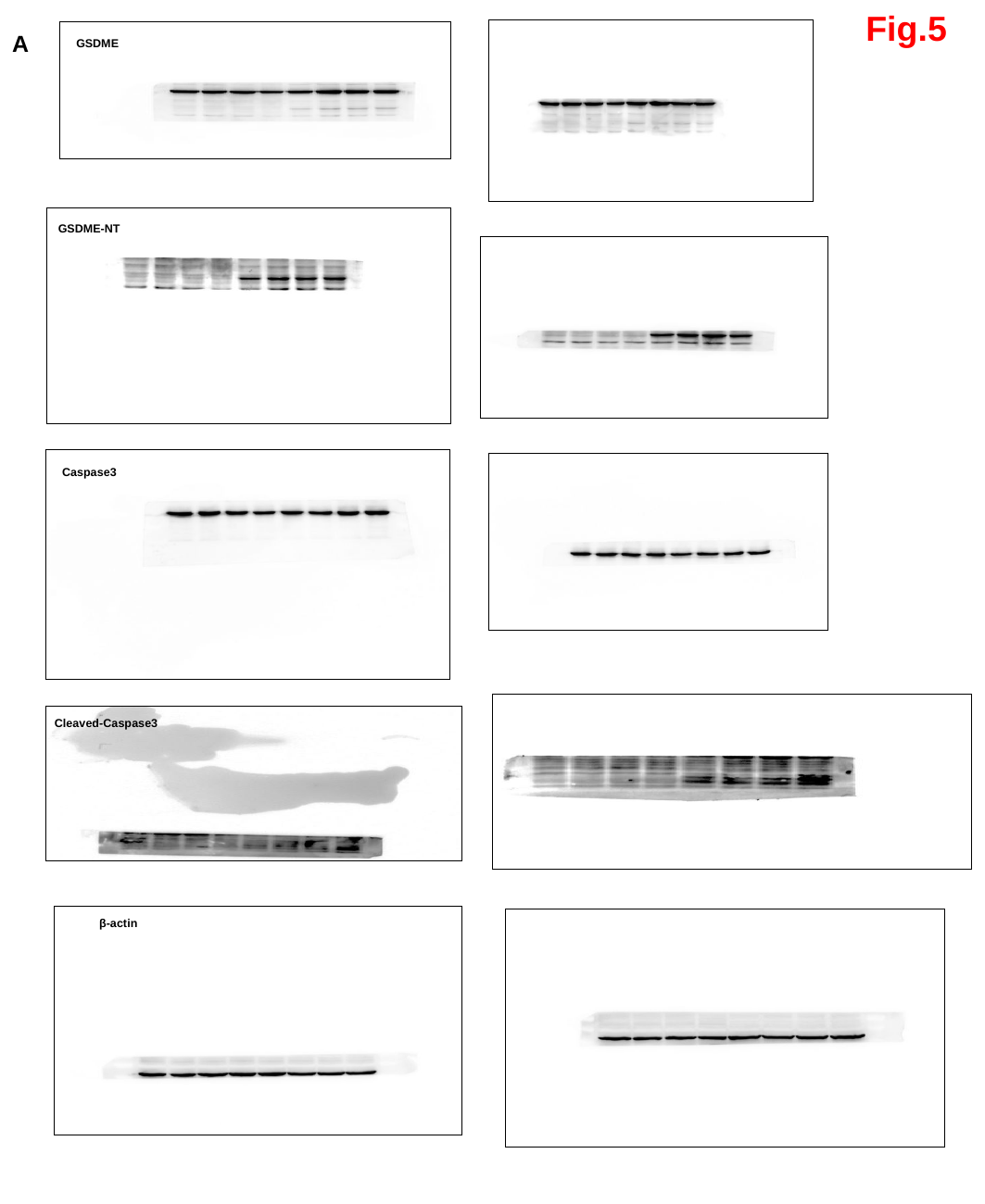

Fig.5
A
GSDME
GSDME-NT
Caspase3
Cleaved-Caspase3
β-actin

## Slide 3
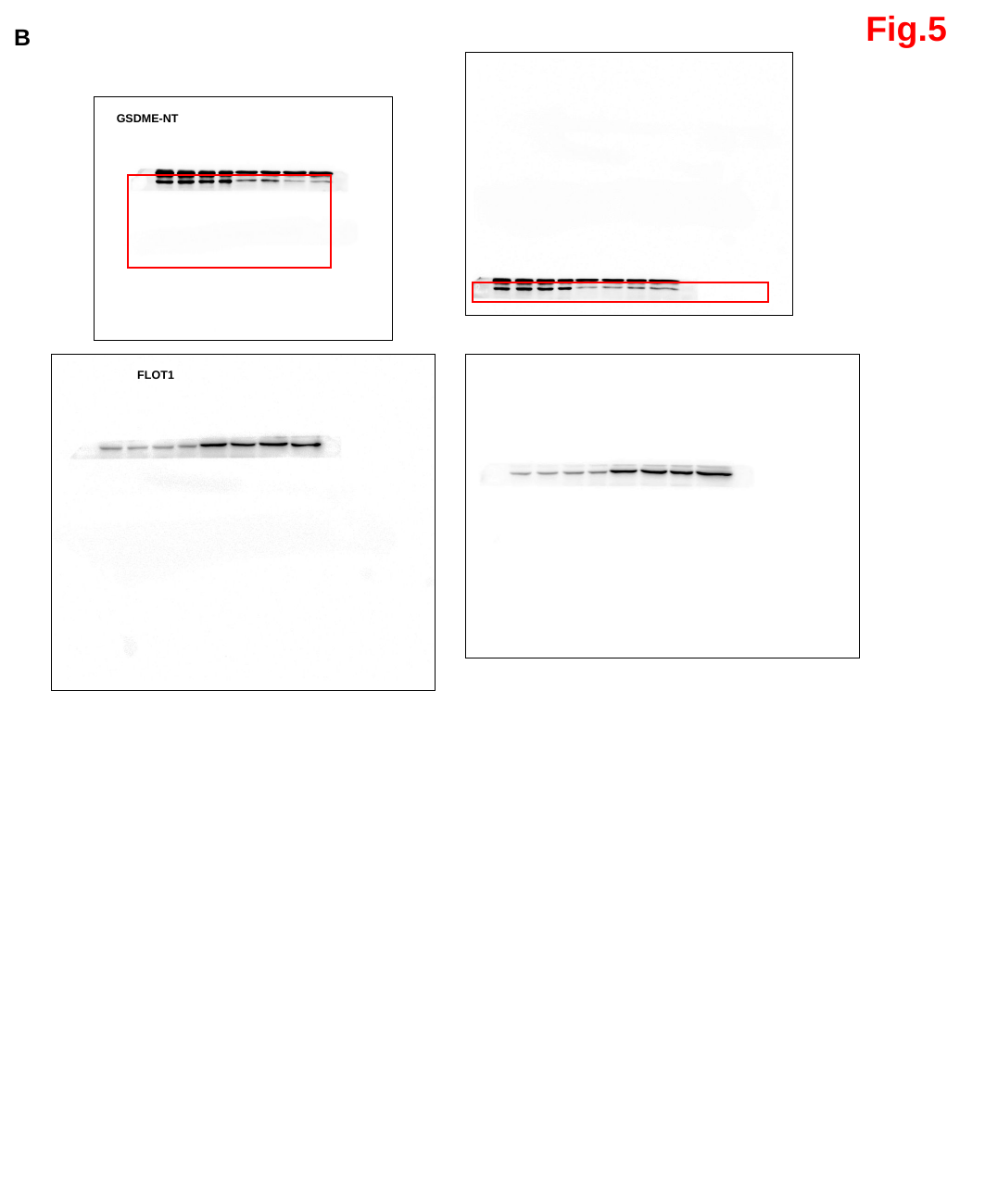

Fig.5
B
GSDME-NT
FLOT1

## Slide 4
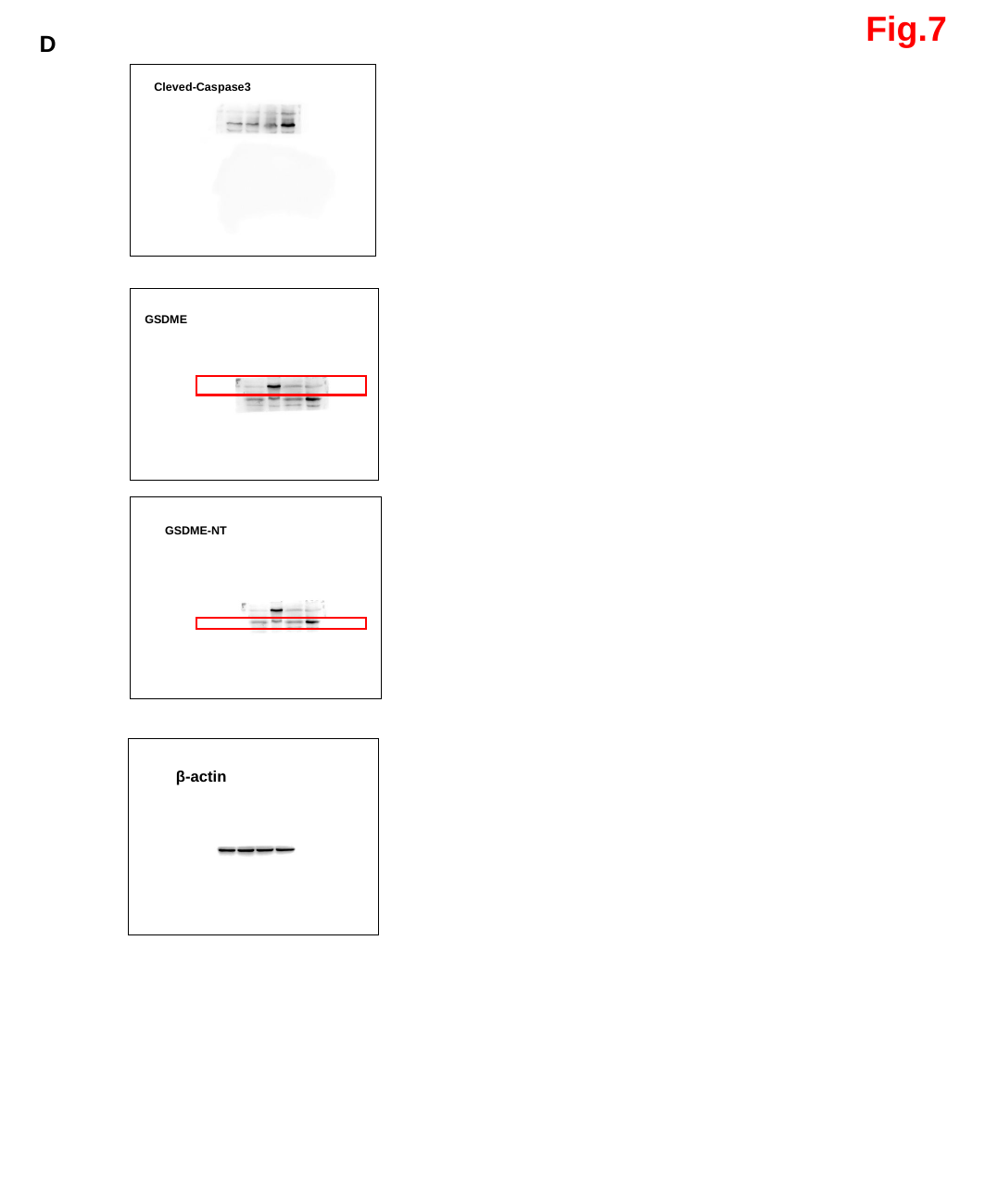

Fig.7
D
Cleved-Caspase3
GSDME
GSDME-NT
β-actin

## Slide 5
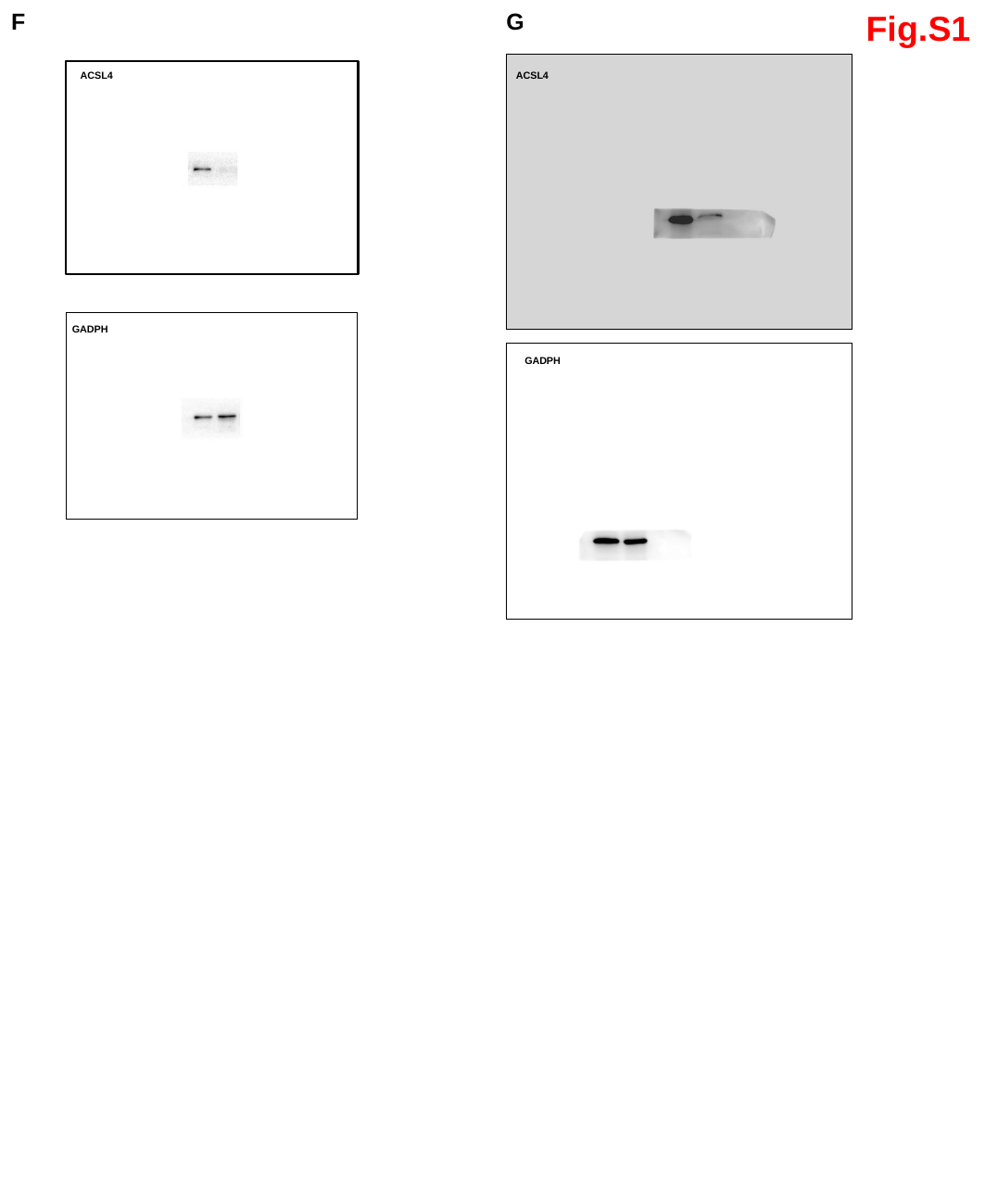

F
G
Fig.S1
ACSL4
ACSL4
GADPH
GADPH

## Slide 6
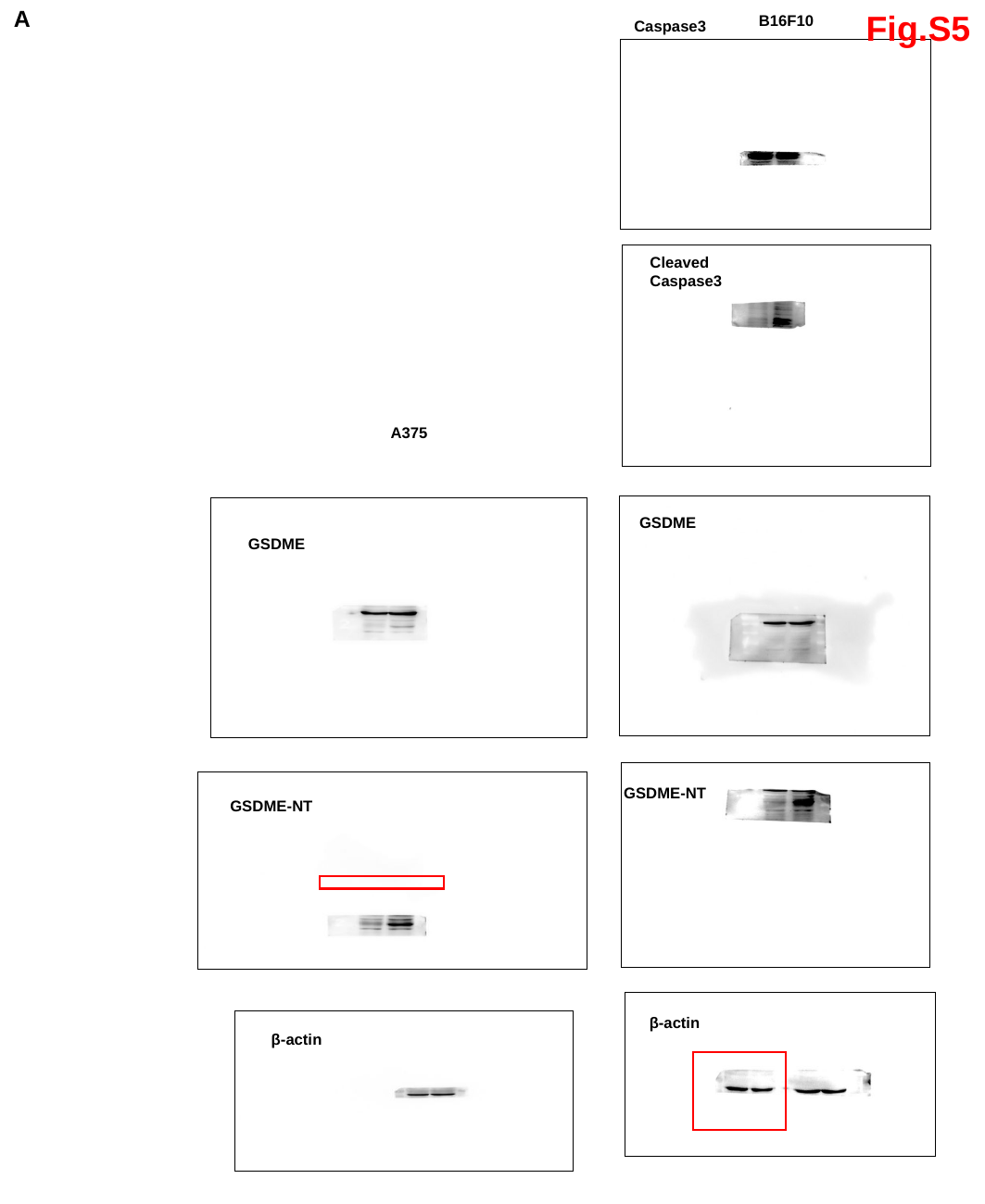

Fig.S5
A
B16F10
Caspase3
Cleaved
Caspase3
A375
GSDME
GSDME
GSDME-NT
GSDME-NT
β-actin
β-actin

## Slide 7
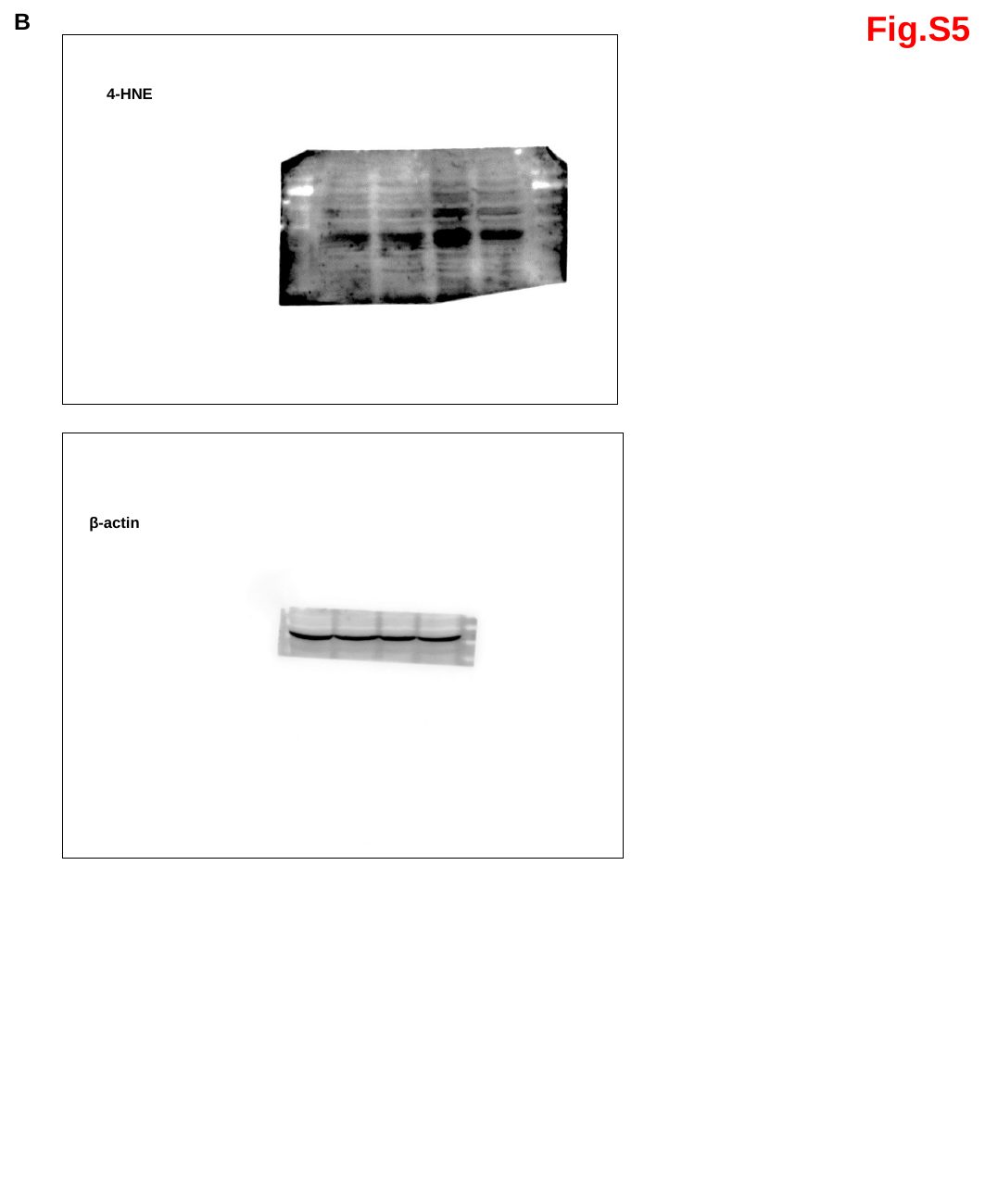

B
Fig.S5
4-HNE
β-actin

## Slide 8
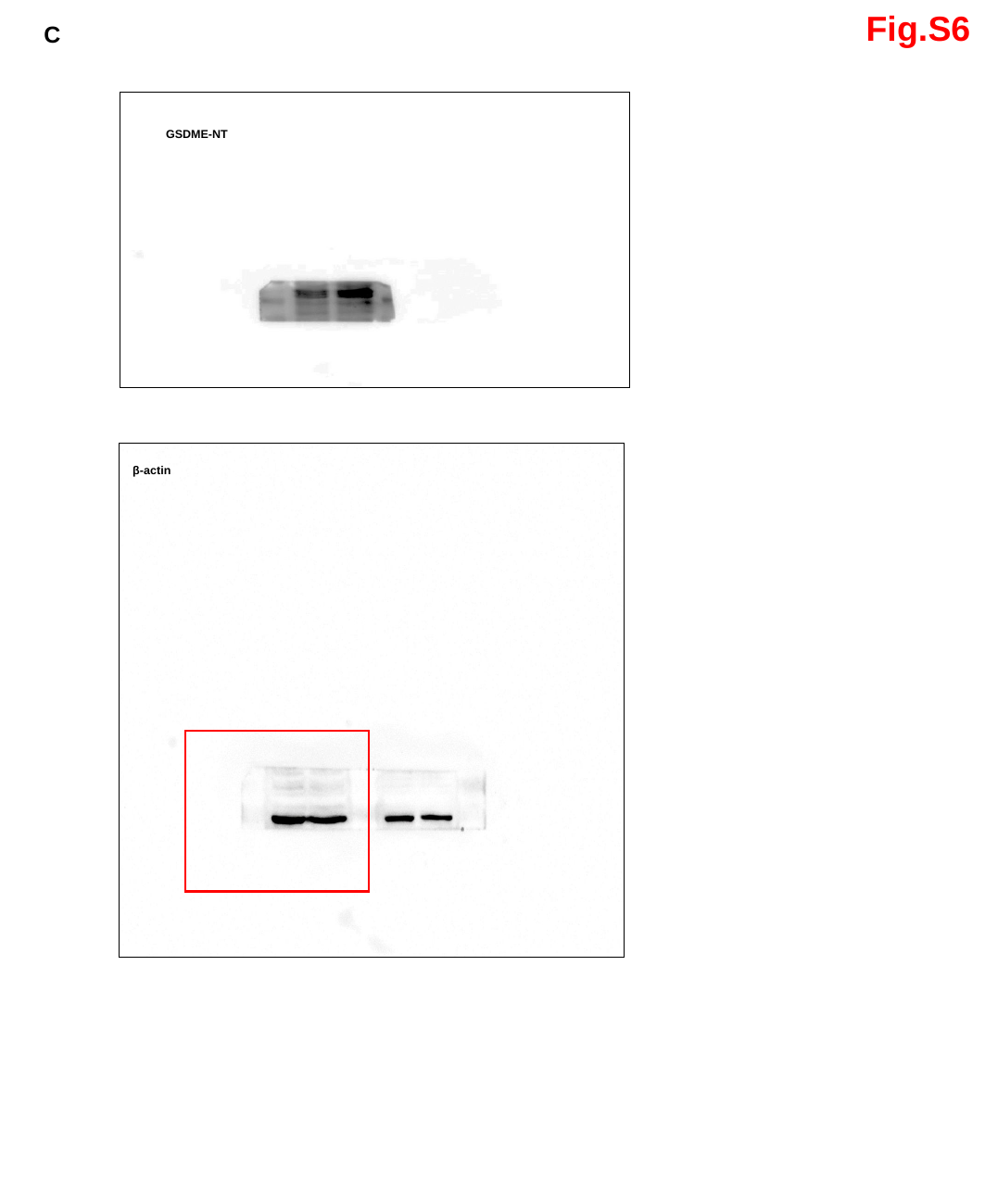

Fig.S6
C
GSDME-NT
β-actin
